# Inhibition of Endoplasmic Reticulum Stress Cooperates with SLC7A11 to Promote Disulfidptosis and Suppress Tumor Growth upon Glucose Limitation

**DOI:** 10.1002/advs.202408789

**Published:** 2024-12-30

**Authors:** Jin Wang, Jing Chen, Kexin Fan, Minglin Wang, Min Gao, Yakun Ren, Shaobo Wu, Qian He, Kangsheng Tu, Qiuran Xu, Yilei Zhang

**Affiliations:** ^1^ Department of Hepatobiliary Surgery the First Affiliated Hospital Department of Biochemistry and Molecular Biology School of Basic Medical Sciences Xi'an Jiaotong University Xi'an Shaanxi 710061 China; ^2^ Key Laboratory of Environment and Genes Related to Diseases of Ministry of Education Xi'an Jiaotong University Xi'an Shaanxi 710061 China; ^3^ Zhejiang Key Laboratory of Tumor Molecular Diagnosis and Individualized Medicine Hangzhou Medical College Hangzhou Zhejiang 311300 China; ^4^ Research Center of Diagnosis and Treatment Technology for Hepatocellular Carcinoma of Zhejiang Province Hangzhou Zhejiang 311300 China; ^5^ Shaanxi Stem Cell Engineering Application Research Center Shaanxi Jiuzhou Biomedical Science and Technology Group Xi'an Shaanxi 710065 China

**Keywords:** disulfidptosis, endoplasmic reticulum stress, glucose deprivation, SLC7A11, tumor

## Abstract

Disulfidptosis is a newly discovered type of regulated cell death triggered by disulfide bond accumulation and NADPH (nicotinamide adenine dinucleotide phosphate) depletion due to glucose deprivation. However, the regulatory mechanisms involving additional cellular circuits remain unclear. Excessive disulfide bond accumulation can impair endoplasmic reticulum (ER) homeostasis and activate the ER stress response. In this study, we found that SLC7A11‐mediated disulfidptosis upon glucose deprivation is accompanied by ER stress induction. Pharmacological inhibition of SLC7A11‐mediated cystine uptake or cystine withdrawal not only blocks disulfidptosis under glucose starvation but also suppresses the ER stress response, indicating a close link between these processes. Moreover, inhibitors targeting the ER stress response promote disulfidptosis, while ER stress inducers suppress glucose starvation‐induced disulfidptosis in SLC7A11‐high‐expressing cells, suggesting a protective role for ER stress during disulfidptosis. Similar effects are observed in cells treated with glucose transporter inhibitors (GLUTi). Finally, combined treatment with ER stress inhibitors and GLUTi significantly suppresses tumor growth both in vitro and in vivo by inducing disulfide stress and subsequent disulfidptosis. In summary, these findings reveal a novel role for ER stress in regulating disulfidptosis and provide theoretical insights into the potential application of GLUTi and ER stress inhibitors in cancer therapy.

## Graphical abstract

Disulfidptosis is a recently identified form of cell death with largely unknown regulatory mechanisms. It is found that SLC7A11‐mediated disulfidptosis in the absence of glucose promotes an ER stress response involving the eIF2α/ATF4 pathway. Inhibition of ER stress enhances tumor suppression by accelerating disulfidptosis induced by GLUT inhibitors, highlighting the potential application of targeting ER stress and disulfidptosis in tumor therapy.

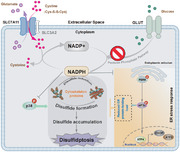

## 1 Introduction

Dysregulation of regulated cell death (RCD) including apoptosis, necroptosis, and ferroptosis is crucial for tumor development. For instance, tumor suppressor p53 and BAP1 could suppress tumor development by promoting either apoptotic or ferroptotic cell death.^[^ ^1^, ^2^, ^3^ ^]^ Meanwhile, tumor cells evolve to bypass the RCD routes which eventually leads to therapy resistance and tumor recurrence.^[^ ^4^ ^]^ Resistance to regulated cell death results in uncontrolled tumor development as well as failure of anti‐cancer treatments such as chemotherapy and radiotherapy.^[^ ^5^ ^]^ Thus, a detailed understanding of the underlying mechanisms and further exploitation of the therapeutic application of RCD for anti‐cancer treatments could improve the efficacy of cancer therapy. Disulfidptosis is a newly discovered type of RCD regulated by the cystine/glutamate antiporter SLC7A11 in the absence of glucose.^[^ ^6^ ^]^ The high level of imported cystine mediated by SLC7A11 requires a large amount of Nicotinamide Adenine Dinucleotide Phosphate (NADPH) to support the reduction of cystine to cysteine. The pentose phosphate pathway (PPP) in glucose metabolism is the major source of intracellular NADPH. As a result, high expression of SLC7A11 promotes glucose dependence in tumor cells.^[^ ^7^ ^]^ Mechanistically, glucose deprivation cuts off NADPH supply and SLC7A11‐mediated cystine uptake keeps NADPH consumption, which drains out NADPH and causes accumulation of intracellular disulfide bonds on cytoskeletal proteins and ultimately, rapid cell death named disulfidptosis.^[^ ^6^ ^]^ Targeting glucose metabolism by glucose transporter inhibitor (GLUTi) is demonstrated as a powerful weapon to induce disulfidptosis and fight against tumors with high‐SLC7A11 expression.^[^ ^6^, ^7^ ^]^ These findings indicate that disulfidptosis might serve as an additional strategy for cancer therapy and the detailed mechanisms underlying disulfidptosis regulation call for further investigation.

The endoplasmic reticulum (ER) is a specialized organelle to maintain cellular homeostasis. It orchestrates the synthesis, folding, and transport of most proteins in eukaryotic cells through various control mechanisms.^[^ ^8^ ^]^ Disulfide bond formation occurs in the ER and promotes structural stability of proteins. Correct disulfide formation requires the assistance of protein disulfide isomerases (PDIs) to introduce disulfides between proximal cysteines and to reduce disulfides for appropriate folding that are not present in the final native structure.^[^ ^9^, ^10^ ^]^ Disturbances in ER homeostasis can cause misfolded proteins, which may eventually lead to unfolded protein response (UPR) or, in other words, ER stress response. To date, various stimuli including energy stress, oxidative stress, nutrient deprivation, and dysregulated calcium levels have been proven to be capable of inducing UPR or ER stress response.^[^ ^11^ ^]^ Interestingly, once activated, ER stress response has a dual role in cell fate control. Initially, ER stress response integrates signaling molecules like PERK/eIF2α to inhibit general protein translation and remove the incompletely unfolded proteins to maintain cell survival. However, if beyond the capacity of re‐establishing protein‐folding homeostasis by ER, cells undergo apoptotic cell death mediated by upregulation of downstream genes of ER stress response like XBP1s, CHOP, etc.^[^ ^12^ ^]^ Thus, the exact modulation of cell death modalities by ER stress under specific conditions should be thoroughly investigated.

Glucose serves as the primary source of energy production and provides varied intermediate metabolites for cell proliferation, and targeting glucose dependency for tumor suppression has been considered a long‐standing strategy to treat tumor patients.^[^ ^13^ ^]^ Previous studies including ours have shown that glucose deprivation dramatically activates ER stress,^[^ ^14^, ^15^, ^16^ ^]^ yet the regulation of ER stress and disulfidptosis in the context of glucose deprivation remains unclear. In this study, our findings revealed that ER stress is closely linked to SLC7A11‐mediated disulfidptosis, and perturbation of ER stress can regulate disulfidptosis. Results in this article will expand the understanding of ER stress in the control of regulated cell death, and open up the potential for new therapeutic modalities in the treatment of tumors.

## 2 Results

### 2.1 SLC7A11 Promotes p38 Phosphorylation During Disulfidptosis Induced by Glucose Deprivation

**Figure 1:**
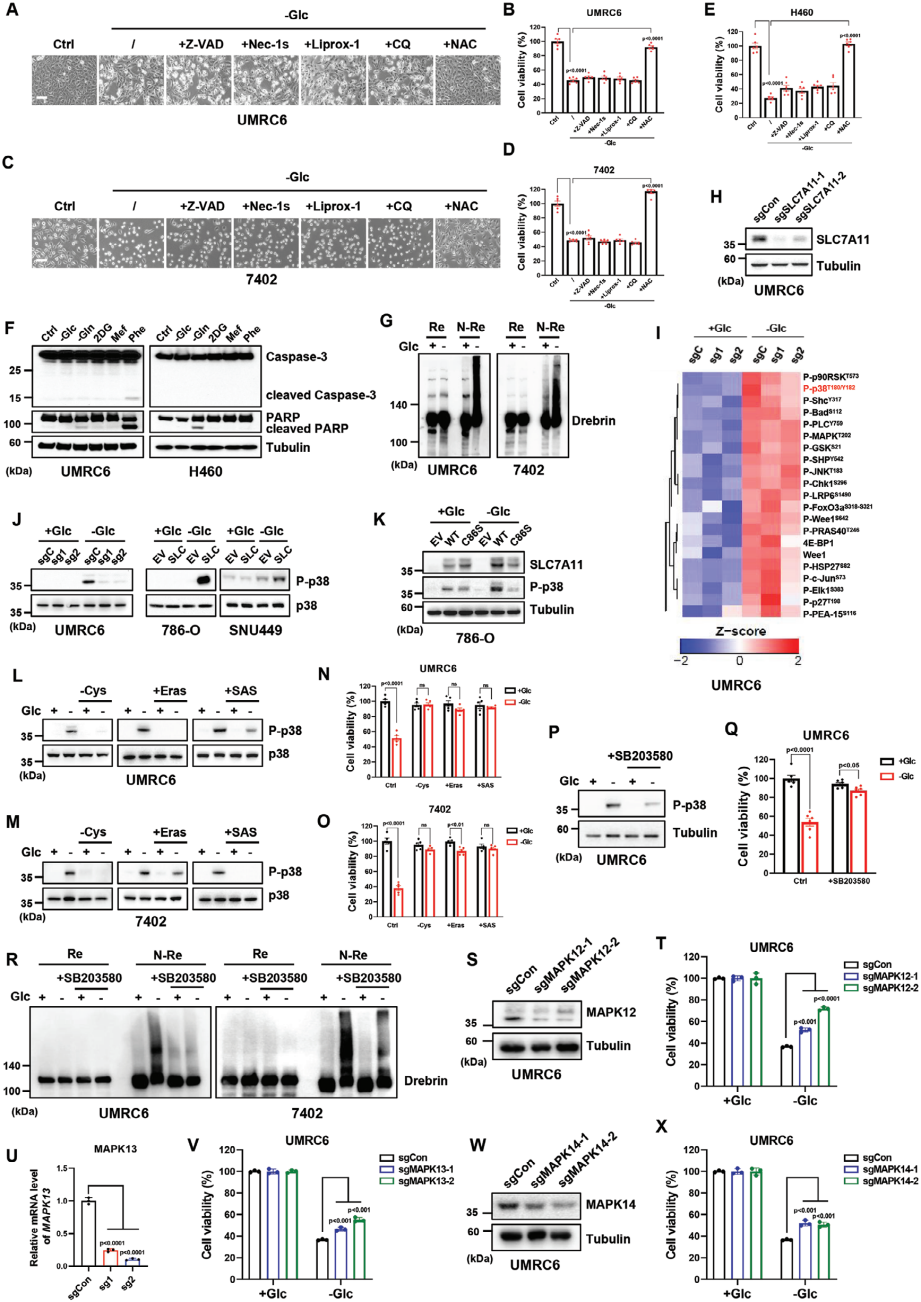
SLC7A11 promotes p38 phosphorylation during disulfidptosis induced by glucose deprivation. A–E) Cell morphological changes (A,C) and cell viability (B,D,E) measured by CCK8 assay in SLC7A11^high^ UMRC6, H460, and 7402 cells cultured in glucose‐containing (Ctrl) or glucose‐free (‐Glc) medium with or without Z‐VAD (5 µm), Nec‐1s (2 µm), Liprox‐1 (5 µm), CQ (20 µm) and NAC (2 mm) for 4–6 h. Scale bars, 100 µm. F) Western blotting analysis of apoptotic markers in UMRC6 and H460 cells cultured in medium without glucose (Glc) or glutamine (Gln) or with energy stress inducers 2‐DG (10 mm), metformin (2 mm) and phenformin (2 mm) for 8 h. G) Reducing and non‐reducing Western blotting analysis of Drebrin in UMRC6 and 7402 cells cultured in glucose‐containing/‐free medium for 4–6 h. H) Western blotting analysis of SLC7A11 expression in control and SLC7A11 knockout UMRC6 cell lines. I) Antibody microarray analysis of involved signaling molecules in sgCon and sgSLC7A11 UMRC6 cells cultured in glucose‐containing/‐free medium. J) Western blotting analysis of p38 phosphorylation of indicated cells cultured in glucose‐containing/‐free medium. K) Western blotting analysis of SLC7A11 expression and p38 phosphoryation in 786‐O empty vector (EV), wild type (WT), and C86S mutant cell lines cultured in glucose‐containing/‐free medium. L,M) Western blotting analysis of p38 phosphoryation in UMRC6 (L) and 7402 (M) cells cultured in glucose‐containing/‐free medium with or without Cystine, Erastin (10 µm) and Sulfasalazine (SAS, 10 µm) for 4–6 h. N,O) Cell viability measured by CCK8 assay in UMRC6 (N) and 7402 (O) cells cultured in glucose‐containing/‐free medium with or without indicated concentrations of Cystine, Erastin and SAS for 4–6 h. P) Western blotting analysis of p38 phosphoryation in UMRC6 cells cultured in glucose‐containing/‐free medium with or without p38 inhibitor SB203580 (50 µm) for 6 h. Q) Cell viability measured by CCK8 assay in UMRC6 cells cultured in glucose‐containing/‐free medium with or without SB203580 treatment. R) Reducing and non‐reducing Western blotting analysis of Drebrin in UMRC6 and 7402 cells cultured in glucose‐containing/‐free medium with or without SB203580 treatment. S–X) Western blotting analysis of MAPK12 (S) and MAPK14 (W) expression, and RT‐PCR analysis of MAPK13 (U) levels in control and corresponding knockout UMRC6 cell lines. Cell viability was measured by CCK8 assay in sgCon and sgMAPK12/13/14 (T/V/X) UMRC6 cells cultured in glucose‐containing/‐free medium. All *p* values were calculated using a two‐tailed unpaired Student's *t*‐test. Data are mean ± SD, *n* ≥ 3 independent repeats unless specified. ns: not significant (*p* > 0.05). All Western blotting was repeated at least twice, independently, with similar results.

Glucose deprivation causes energy stress and eventually induces cell death with distinct mechanisms in tumor cells. Previously, we have demonstrated that SLC7A11 promotes glucose dependency, and high expression of SLC7A11 is a major contributor to triggering disulfidptosis in tumor cells.^[^ ^6^ ^]^ To further study the mechanistic regulation of disulfidptosis by SLC7A11, we assessed the expression levels of SLC7A11 in several human cancer cell lines (Figure S1A, Supporting Information). Rapid cell death was observed in UMRC6, 7402 and H460 cells with high expression of SLC7A11 in the absence of glucose, while only N‐acetyl‐cysteine (NAC) rather than cell death inhibitors such as Z‐VAD‐FMK (apoptosis inhibitor), Nec‐1s (necroptosis inhibitor), Liproxstain‐1 (Liprox‐1, ferroptosis inhibitor), or chloroquine (CQ, autophagy inhibitor) significantly rescued cell death caused by glucose starvation (**Figure** **1**A–E). A similar observation was found in the SLC7A11‐overexpressed SNU449, RCC4, and 786‐O cell lines, but not in their control cell lines with low expression of SLC7A11 (Figure S1B–G, Supporting Information). Glucose deprivation predominantly induces energy stress and apoptotic cell death. Next, we used different energy stress inducers to treat SLC7A11‐high tumor cells and found that glutamine (Gln) starvation could induce cleavage of PARP in UMRC6 and H460 cells, and phenformin (Phe) could induce cleavage of caspase‐3 and PARP in UMRC6 cells (Figure 1F). In addition, metformin and phenformin induced significant cell death, disulfidptosis inhibitors like 2‐DG and D‐ or L‐penicillamine failed to prevent this outcome, supporting the idea that glucose deprivation and metformin or phenformin elicit distinct forms of cell death, despite both causing energy stress (Figure S1H, Supporting Information). Staurosporine (STS) is a known apoptosis inducer and could induce cleaved caspase‐3 in cells regardless of SLC7A11 level (Figure S1I, Supporting Information). However, glucose starvation failed to induce the cleavage of caspase‐3 in these cells (Figure 1F; Figure S1I, Supporting Information), indicating that neither energy stress nor apoptotic cell death is involved in the regulation of cell survival in SLC7A11‐high tumor cells upon glucose deprivation.

Disulfidptosis is characterized by increased disulfide bonds within cytoskeletal proteins such as Drebrin, MYH9 and FLNA/B, which could be monitored by electrophoretic mobility shift under non‐reducing conditions.^[^ ^6^ ^]^ Indeed, glucose starvation resulted in obvious migration retardation of Drebrin in SLC7A11‐high (UMRC6 and 7402) or SLC7A11‐overexpressed (SNU449‐SLC and RCC4‐SLC) cells (Figure 1G; Figure S1J, Supporting Information). To explore the involved signaling molecules during disulfidptosis, an antibody microarray was applied to determine the molecules with altered phosphorylation levels in UMRC6 control (sgC) and SLC7A11‐knockout (sg1 and sg2) cell lines (Figure 1H,I). As shown in Figure 1I, the phosphorylation level of p38 is the most dramatically upregulated molecule upon glucose deprivation in UMRC6‐sgC cells, while knockout of SLC7A11 significantly abolished this effect. Additional results further confirmed that high expression of SLC7A11 significantly promotes phosphorylation of p38 upon glucose starvation, while knockout of SLC7A11 expression does the opposite, suggesting that p38 phosphorylation is regulated by SLC7A11 level and glucose availability (Figure 1J). To verify the role of SLC7A11‐mediated cystine uptake in the regulation of p38 phosphorylation, we generated a SLC7A11 mutant C86S, which severely lost its ability to import cystine,^[^ ^17^ ^]^ and found that its ability to promote p38 phosphorylation duringe starvation was significantly lower than that of SLC7A11 WT (Figure 1K). In addition, either cystine starvation or inhibition of SLC7A11 by erastin and sulfasalazine (SAS) significantly reduced glucose starvation‐induced p38 phosphorylation in SLC7A11‐high cell lines (Figure 1L,M; Figure S1K, Supporting Information). Since the withdrawal of cystine or inhibition of SLC7A11 also significantly suppressed glucose deprivation‐induced disulfidptosis (Figure 1N,O; Figure S1L,M, Supporting Information), it prompted us to study the role of p38 in disulfidptosis regulation. Notably, the p38 inhibitor SB203580, which suppressed glucose starvation‐induced p38 phosphorylation, successfully inhibited disulfidptosis and prevented migration retardation of Drebrin in SLC7A11‐high tumor cells in the absence of glucose (Figure 1P–R; Figure S1N–Q, Supporting Information). The p38 MAPK pathway is well known for its role in environment stress signal transduction. Although p38 is involved in a wide range of cellular activities, its regulatory mechanisms at the molecular level are still poorly understood. There are four isoforms of p38 MAPK, which are encoded by distinct genes: p38α (MAPK14); p38β (MAPK11); p38γ (MAPK12) and p38δ (MAPK13).^[^ ^18^ ^]^ Thus, we constructed individual sgRNA in a CRISPR‐V2/Cas9 vector specifically targeting the p38 isoforms mentioned above, and generated corresponding gene knockout cell lines. Except for MAPK11, knockout of other p38 isoforms (MAPK12/13/14) indeed significantly restored cell viability upon glucose deprivation to varying degrees (Figure 1S–X; Figure S1R,S, Supporting Information), suggesting an overlapped function of p38 isoforms in regulating disulfidptosis. These results suggest that p38 activation is involved in the regulation of disulfidptosis, though the detailed mechanism requires further investigation.

### 2.2 Glucose Deprivation Induces Disulfidptosis Independent of Mito‐ROS and GSH

**Figure 2:**
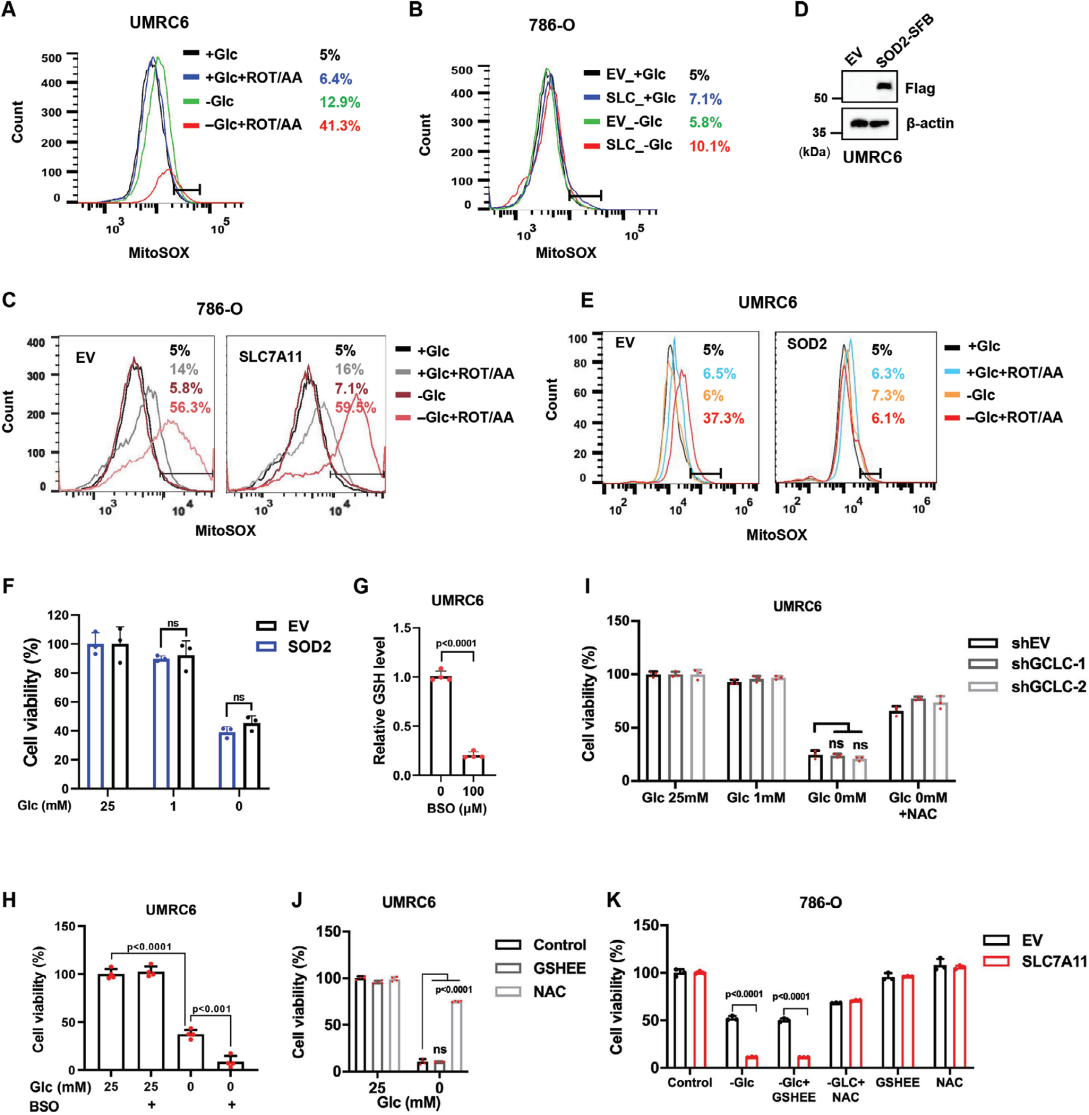
Glucose deprivation induces disulfidptosis independent of mito‐ROS and GSH. A–C) Flow cytometry analysis of Mito‐ROS levels in UMRC6 cells (A) or empty vector (EV) and SLC7A11 overexpressing (SLC) 786‐O cells (B,C) cultured in glucose‐containing/‐free medium with or without Rotenone/Antimycin A1 (ROT/AA: 0.5 µm/0.1 mm) for 4–6 h. D) Western blotting analysis of SOD2 expression in EV and SOD2 overexpressing UMRC6 cell lines. E,F) Mito‐ROS level measurement by flow cytometry (E) and cell viability measurement by CCK8 assay (F) in EV and SOD2 overexpressing UMRC6 cells cultured in glucose‐containing/‐free medium with or without Rotenone/Antimycin A1 for 4–6 h. G) Relative GSH levels of UMRC6 cells treated with indicated concentrations of BSO. H) Cell viability measured by CCK8 assay in UMRC6 cells cultured in glucose‐containing/‐free medium with or without BSO treatment. I) Cell viability was measured by CCK8 assay in EV and GCLC knockdown UMRC6 cells cultured in a medium with different concentrations of glucose with or without NAC treatment (2 mm). J,K) Cell viability measured by CCK8 assay in UMRC6 cells (J) or EV and SLC7A11 overexpressing 786‐O cells K) cultured in glucose‐containing/‐free medium with or without GSHEE (200 µm) and NAC. All *p* values were calculated using a two‐tailed unpaired Student's *t*‐test. Data are mean ± SD, *n* ≥ 3 independent repeats. ns: not significant (*p* > 0.05).

Glucose starvation limits the supply to the electron transport chain (ETC), inhibition of which subsequently generates mitochondrial superoxide (mito‐ROS) to induce oxidative stress and cell death.^[^ ^19^, ^20^ ^]^ We found that treatment with glucose starvation or ETC inhibitors rotenone and antimycin A (ROT/AA) had a very limited effect in terms of inducing mito‐ROS, as indicated by a specific dye MitoSOX (**Figure** **2**A–C). However, the combination of the above treatments greatly increased the mito‐ROS levels in tumor cells regardless of SLCA11 expression level (Figure 2A–C). This phenomenon was also observed in 786‐O control (EV) and SLC7A11‐overexpressing (SLC) cells (Figure 2B,C). Superoxide dismutase 2 (SOD2) is known to effectively eliminate mito‐ROS in mitochondria.^[^ ^21^, ^22^ ^]^ We constructed a SOD2‐overexpressed UMRC6 cell line, and found that SOD2 couldn't rescue cell death induced by glucose starvation despite the fact that SOD2 almost completely inhibited the upregulation of mito‐ROS levels induced by combined treatments with glucose starvation and ROT/AA (Figure 2D–F; Figure S2A, Supporting Information). In addition, the mitochondria‐specific antioxidant mito‐CoQ10 (MQ) did not prevent the disulfidptosis of UMRC6 cells under glucose deprivation conditions, though MQ partially suppressed cell death caused by tert‐Butyl hydroperoxide (TBH), which mildly induces oxidative stress within cells (Figure S2B–D, Supporting Information). These results suggest that triggering disulfidptosis by glucose starvation is independent of mitochondrial superoxide. Knockout of SLC7A11 significantly reduced the uptake of extracellular cystine by UMRC6 cells (Figure S2E, Supporting Information), which caused the downregulation of intracellular reduced glutathione (GSH) levels within cells, similar to the effect of glucose starvation alone (Figure S2F, Supporting Information). However, there was no significant difference in GSH levels between control and SLC7A11‐knockout cells under glucose starvation conditions (Figure S2F, Supporting Information), implying that SLC7A11 rarely affects cellular GSH levels upon glucose starvation. To further explore the role of GSH in the regulation of disulfidptosis, we used buthionine sulfoximine (BSO, an inhibitor of γ‐glutamylcysteine synthetase) to block GSH synthesis, and found that BSO treatment significantly reduced GSH levels in UMRC6 cells (Figure 2G). But, unlike cystine deprivation or SLC7A11 inhibition which is also capable of decreasing GSH levels, BSO failed to prevent disulfidptosis upon glucose starvation (Figure 2H). In addition, the knockdown of glutamate‐cysteine ligase catalytic subunit (GCLC) in UMRC6 cells which suppressed intracellular GSH synthesis as we previously reported,^[^ ^23^ ^]^ couldn't inhibit glucose deprivation‐induced disulfidptosis either (Figure 2I; Figure S2G, Supporting Information). To mimic the SLC7A11‐overexpressed cells with upregulated GSH levels, a GSH derivate glutathione ethyl ester (GSHEE) that replenishes the intracellular GSH level was used to treat cells with or without glucose, and the results showed that GSHEE didn't regulate cell death upon glucose starvation (Figure 2J,K; Figure S2H, Supporting Information). Taken together, our data suggest that glucose deprivation induces disulfidptosis in SLC7A11‐high cells independent of mitochondrial superoxide and GSH.

### 2.3 ER Stress Response is Activated During Disulfidptosis Caused by SLC7A11 and Glucose Deprivation

**Figure 3:**
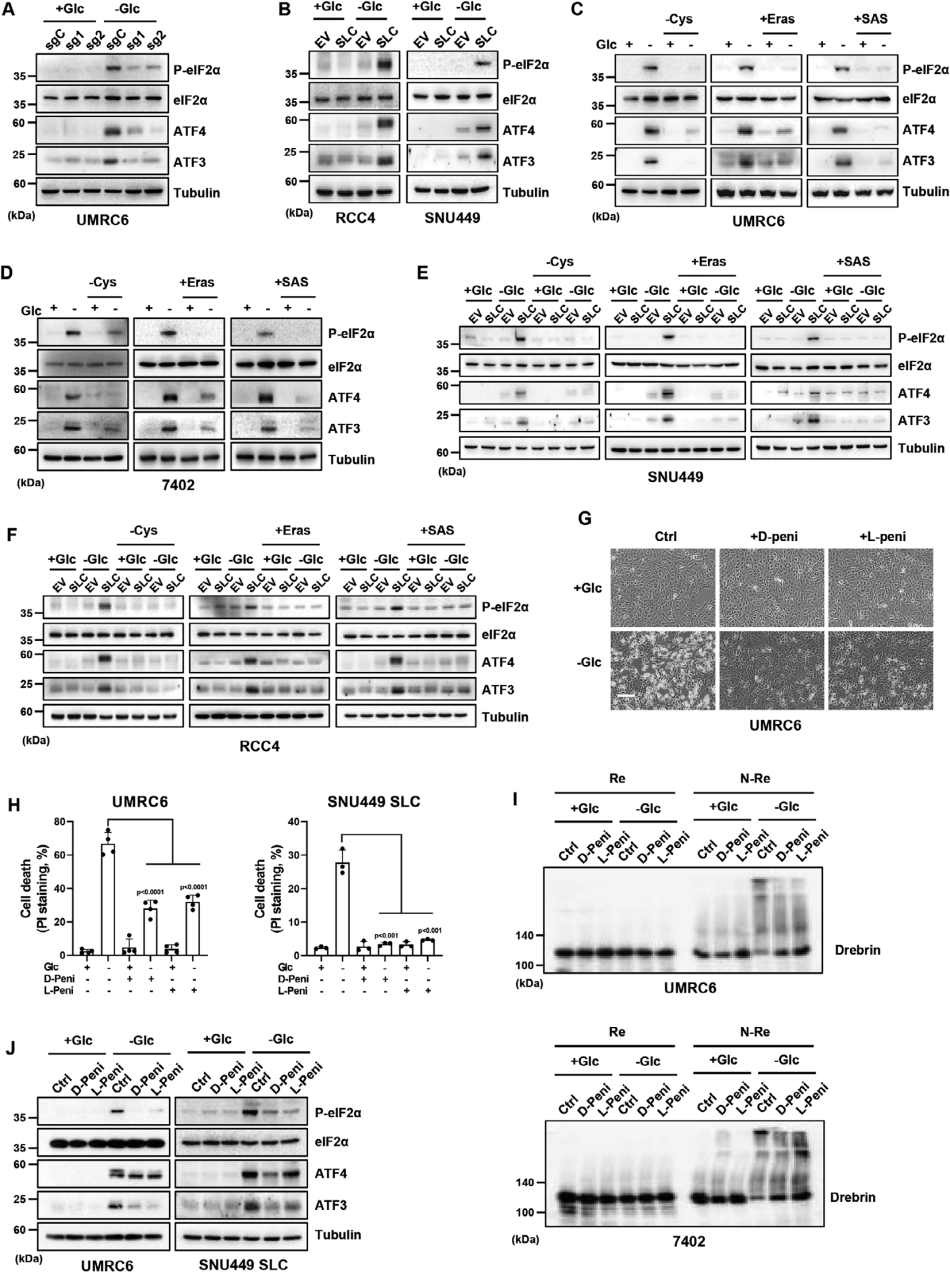
The ER stress response is activated during disulfidptosis caused by SLC7A11 and glucose deprivation. A,B) Western blotting analysis of PERK/eIF2α/ATF4 pathway proteins in control and SLC7A11 knockout UMRC6 cells (A) or in EV and SLC7A11 overexpressing RCC4 and SNU449 cells (B) cultured in glucose‐containing/‐free medium. C–F) Western blotting analysis of PERK/eIF2α/ATF4 pathway proteins in UMRC6 cells (C), 7402 cells (D) or in EV and SLC7A11 overexpressing SNU449 (E) and RCC4 cells (F) cultured in glucose‐containing/‐free medium with or without Cystine, Erastin and Sulfasalazine treatment. G) Cell morphological changes in UMRC6 cells cultured in glucose‐containing/‐free medium with or without D‐/L‐penicillamine (1 mm) treatment. Scale bars, 100 µm. H) Cell death measured by PI staining in UMRC6 and SNU449‐SLC cells cultured in glucose‐containing/‐free medium with or without D‐/L‐penicillamine. I) Reducing and non‐reducing Western blotting analysis of Drebrin in UMRC6 and 7402 cells cultured in glucose‐containing/‐free medium with or without D‐/L‐penicillamine. J) Western blotting analysis of PERK/eIF2α/ATF4 pathway proteins in UMRC6 and SNU449 SLC cells cultured in glucose‐containing/‐free medium with or without D‐/L‐penicillamine. All *p* values were calculated using a two‐tailed unpaired Student's *t*‐test. Data are mean ± SD, *n* ≥ 3 independent repeats. All Western blotting was repeated at least twice, independently, with similar results.

The accumulation of unfolded proteins within endoplasmic reticulum (ER) activates ER stress response to restore ER homeostasis. Our previous report demonstrated that glucose starvation promotes ER stress response, but whether it's regulated by SLC7A11 and cystine metabolism remains unclear.^[^ ^24^ ^]^ ER stress response or unfolded protein response mainly activates three downstream pathways including PERK/eIF2α, XBP1s or JNK to orchestrate the regulation of gene transcription and/or protein translation (Figure S3A, Supporting Information). We found that glucose starvation upregulates P‐JNK and XBP1s in a SLC7A11‐independent manner, while NRF2 was consistently upregulated in SLC7A11‐high cells, suggesting the induction of oxidative stress upon glucose deprivation within these cells (Figure S3B, Supporting Information). Notably, knockout of SLC7A11 attenuated the upregulation of P‐eIF2α, ATF4, and ATF3 caused by glucose starvation, while overexpression of SLC7A11 boosted their levels under the same condition (**Figure** **3**A,B). Furthermore, the removal of extracellular cystine or inhibition of SLC7A11‐mediated cystine uptake by erastin or SAS dramatically abolished the upregulation of P‐eIF2α, ATF4, and ATF3 caused by SLC7A11 and glucose starvation (Figure 3C–F). Next, to figure out whether these ER stress‐responsive genes are regulated by disulfidptosis, we employed penicillamine (including both D‐ and L‐ penicillamine) which prevents the accumulation of intracellular disulfide through disulfide exchange to inhibit disuldfiptosis and relieve migration retardation of Drebrin as shown in Figure 3G,I and Figure S3C (Supporting Information), and found that the upregulation of P‐eIF2α, ATF4, and ATF3 caused by glucose starvation was largely abolished in SLC7A11‐high or overexpressed tumor cells (Figure 3J; Figure S3D, Supporting Information), indicating that ER stress response is closely involved during disulfidptosis. To explore the underlying link between glucose metabolism and ER stress response, we conducted correlation analysis on the expression levels between ATF3 and glucose metabolism‐related genes in datasets from The Cancer Genome Atlas (TCGA), and found that ATF3 was positively correlated with the glucose transporter SLC2A3 in most tumor types. However, the levels of pentose phosphate pathway (PPP) genes like G6PD, PGD, TALDO1 or TKT exhibited a negative correlation with that of ATF3 in a large number of tumors (Figure S3E, Supporting Information). The PPP pathway is critical for NADPH generation and redox homeostasis, which ultimately contributes to disulfidptosis regulation. Collectively, the above results prompted us to further study the role of ER stress in the modulation of disulfidptosis.

### 2.4 Inhibition of ER Stress Response Promotes Disulfidptosis

**Figure 4:**
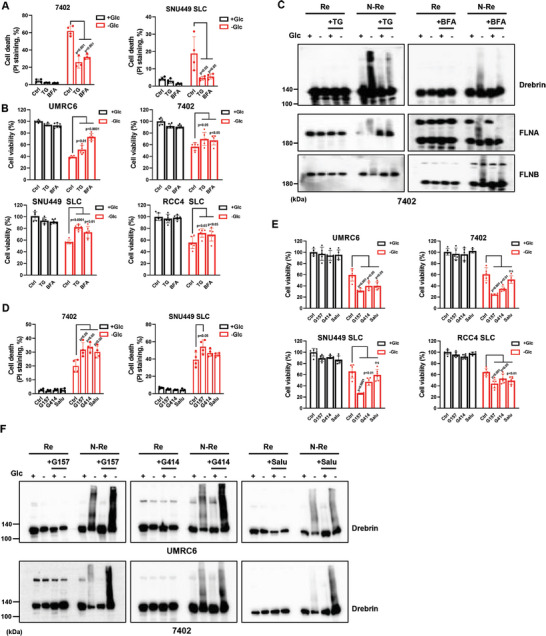
Inhibition of ER stress response promotes disulfidptosis. A,B) Cell death measured by PI staining (A) and cell viability measured by CCK8 assay (B) in SLC7A11 high expressing cells cultured in glucose‐containing/‐free medium with or without ER stress response inducers Thapsigargin (TG, 2 µm) and Brefeldin A (BFA, 5 µm). C) Reducing and non‐reducing Western blotting analysis of cytoskeletal proteins in 7402 cells cultured in glucose‐containing/‐free medium with or without TG and BFA. D,E) Cell death measured by PI staining (D) and cell viability measured by CCK8 assay (E) in SLC7A11 high expressing cells cultured in glucose‐containing/‐free medium with or without ER stress response inhibitors GSK2656157 (G157, 10 µm), GSK2606414 (G414, 10 µm) and Salubrinal (Salu, 20 µm). F) Reducing and non‐reducing Western blotting analysis of Drebrin in UMRC6 and 7402 cells cultured in glucose‐containing/‐free medium with or without G157, G414, and Salu. All *p* values were calculated using a two‐tailed unpaired Student's *t*‐test. Data are mean ± SD, *n* ≥ 3 independent repeats. ns: not significant (*p* > 0.05). All Western blotting was repeated at least twice, independently, with similar results.

To investigate the regulation of disulfidptosis by ER stress, we first used known ER stress inducers including thapsigargin (TG) and Brefeldin A (BFA) to treat cells with or without glucose and found that TG or BFA could accelerate ER stress response like P‐eIF2α/ATF4/ATF3 pathway in the absence of glucose as expected (Figure S4A,B, Supporting Information). Meanwhile, these ER stress inducers at least partially restore the cell viability upon glucose starvation in SLC7A11‐high cells including UMRC6, 7402, as well as SLC7A11‐overpressed RCC4 and SNU449 cells (**Figure** **4**A,B). Consistently, TG or BFA also reduced the migration retardation of cytoskeletal proteins such as Drebrin, FLNA, or FLNB caused by glucose starvation‐induced formation of disulfide bonds (Figure 4C). However, similar to previous reports,^[^ ^25^, ^26^, ^27^ ^]^ we noted that treatment with ER stress inducers such as TG, BFA, and tunicamycin (TUN) individually could induce cleaved PARP (Figure S4C, Supporting Information), suggesting the pro‐apoptotic effects of ER stress response is not involved in its suppressive effect on disulfidptosis. GSK2606414 (G414) and GSK2656157 (G157) are selective PERK inhibitors,^[^ ^28^, ^29^ ^]^ and Salubrinal (Salu) is a selective inhibitor of eIF2α dephosphorylation.^[^ ^30^ ^]^ These ER stress inhibitors significantly inhibited the upregulation of ER stress response genes like ATF4 and ATF3 due to glucose starvation in UMRC6 and 7402 cells (Figure S4D,E, Supporting Information). Consequently, inhibition of ER stress response significantly decreased cell viability in SLC7A11‐high cells under glucose deprivation conditions (Figure 4D,E). Notably, the ER stress inhibitors dramatically slow the migration of cytoskeletal proteins under non‐reducing conditions (Figure 4F; Figure S4F,G, Supporting Information). In summary, these results demonstrate the protective role of ER stress response in SLC7A11‐mediated disulfidptosis upon glucose starvation.

### 2.5 ER Stress Response Suppresses Disulfidptosis in a NADPH and ROS‐Independent Manner

**Figure 5:**
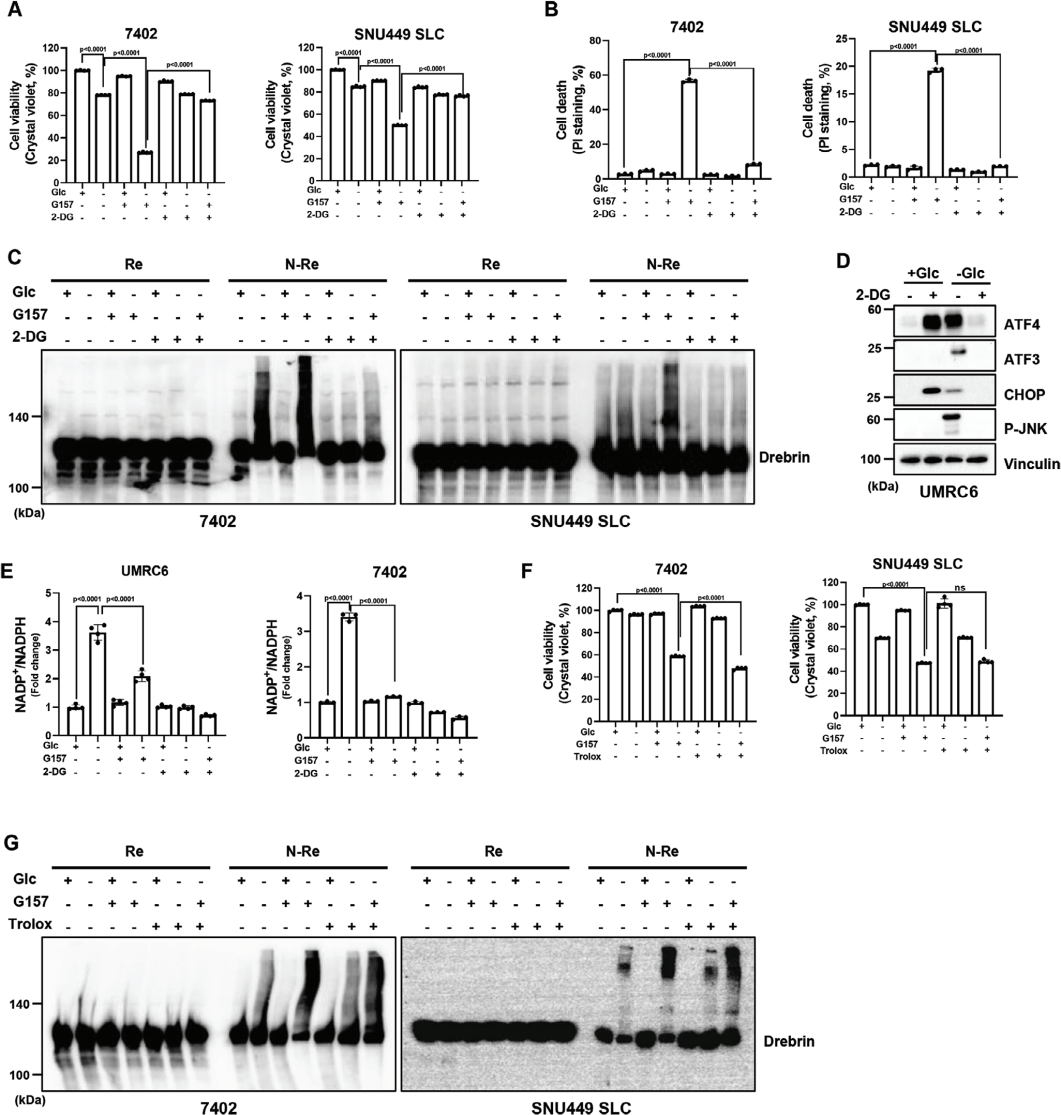
ER stress response suppresses disulfidptosis in a NADPH and ROS‐independent manner. A,B) Cell viability measured by CCK8 assay (A) and cell death measured by PI staining (B) in 7402 and SNU449‐SLC cells cultured in glucose‐containing/‐free medium with or without G157 and 2‐DG. C) Reducing and non‐reducing Western blotting analysis of Drebrin in 7402 and SNU449‐SLC cells cultured in glucose‐containing/‐free medium with or without G157 and 2‐DG. D) Western blotting analysis of ER stress response signaling proteins in UMRC6 cells cultured in glucose‐containing/‐free medium with or without 2‐DG. E) NADP^+^/NADPH ratios of UMRC6 and 7402 cells cultured in glucose‐containing/‐free medium with or without G157 and 2‐DG. F) Cell viability measured by CCK8 assay in 7402 and SNU449‐SLC cells cultured in glucose‐containing/‐free medium with or without G157 and Trolox (2 mm). G) Reducing and non‐reducing Western blotting analysis of Drebrin in 7402 and SNU449‐SLC cells cultured in glucose‐containing/‐free medium with or without G157 and Trolox. All *P* values were calculated using a two‐tailed unpaired Student's *t*‐test. Data are mean ± SD, *n* ≥ 3 independent repeats. ns: not significant (*p* > 0.05). All Western blotting was repeated at least twice, independently, with similar results.

Studies have confirmed that SLC7A11‐mediated disulfidptosis upon glucose deprivation can be rescued by 2‐deoxyglucose (2‐DG), a glucose analog that promotes NADPH generation through the PPP pathway.^[^ ^6^, ^7^ ^]^ Besides, 2‐DG also completely inhibited glucose starvation‐induced ER stress response including the upregulation of ATF4, ATF3, CHOP, and P‐JNK. However, it could upregulate ATF4 and CHOP in the presence of glucose, indicating a distinct mechanism underlying the regulation of ATF4/CHOP by 2‐DG alone (**Figure** **5**D).^[^ ^31^ ^]^ We found that ER stress inhibitor G157 significantly decreased cell viability and boosted disulfidptosis in the absence of glucose, which was almost completely reversed by 2‐DG (Figure 5A,B; Figure S5A, Supporting Information). Besides, treatment with 2‐DG largely abrogated G157‐enhanced band retardation of Drebrin under non‐reducing conditions upon glucose starvation in SLC7A11‐high cells (Figure 5C), suggesting NADPH supplement successfully suppresses disulfidptosis stimulated by glucose deprivation and ER stress inhibition. Next, we wondered whether the enhanced effect of G157 on disulfidptosis was achieved by increasing NADP^+^/NADPH ratio. Interestingly, we observed that G157 significantly reduced the glucose starvation‐induced upregulation of NADP^+^/NADPH ratio in UMRC6 and 7402 cells, while 2‐DG decreased the ratio of NADP^+^/NADPH as expected (Figure 5E). Nevertheless, the ER stress inducers like TG and BFA didn't affect NADP^+^/NADPH ratio under normal or glucose deprivation conditions (Figure S5B, Supporting Information), suggesting the regulation of disulfidptosis by ER stress is independent of NADPH level. Since NADPH is important for maintaining redox homeostasis, glucose starvation‐caused NADPH depletion in SLC7A11‐high cells inevitably induced oxidative stress as shown in Figure S5C,D (Supporting Information). Thus, we treated the cells with antioxidant Trolox and found that treatment with Trolox could efficiently bring down the level of reactive oxygen species (ROS) induced by glucose deprivation and G157 (Figure S5E,F, Supporting Information). However, Trolox neither restored the cell viability decreased by glucose deprivation and G157, nor did it prevent the band retardation of Drebrin due to the accumulation of disulfide bonds during disulfidptosis (Figure 5F,G; Figure S5G, Supporting Information). Meanwhile, we noticed that antioxidants such as Trolox and TEMPO failed to suppress glucose starvation‐stimulated ER stress response (Figure S5H, Supporting Information), suggesting ER stress response is activated by glucose deprivation in a ROS‐independent manner during disulfidptosis. Taken together, these findings indicate that inhibition of ER stress response promotes disulfidpotosis possibly not through regulating NADPH and ROS levels.

### 2.6 Inhibition of ER Stress Collaborates with GLUT Inhibitor to Promote Disulfidptosis and Suppress Tumor Growth

**Figure 6:**
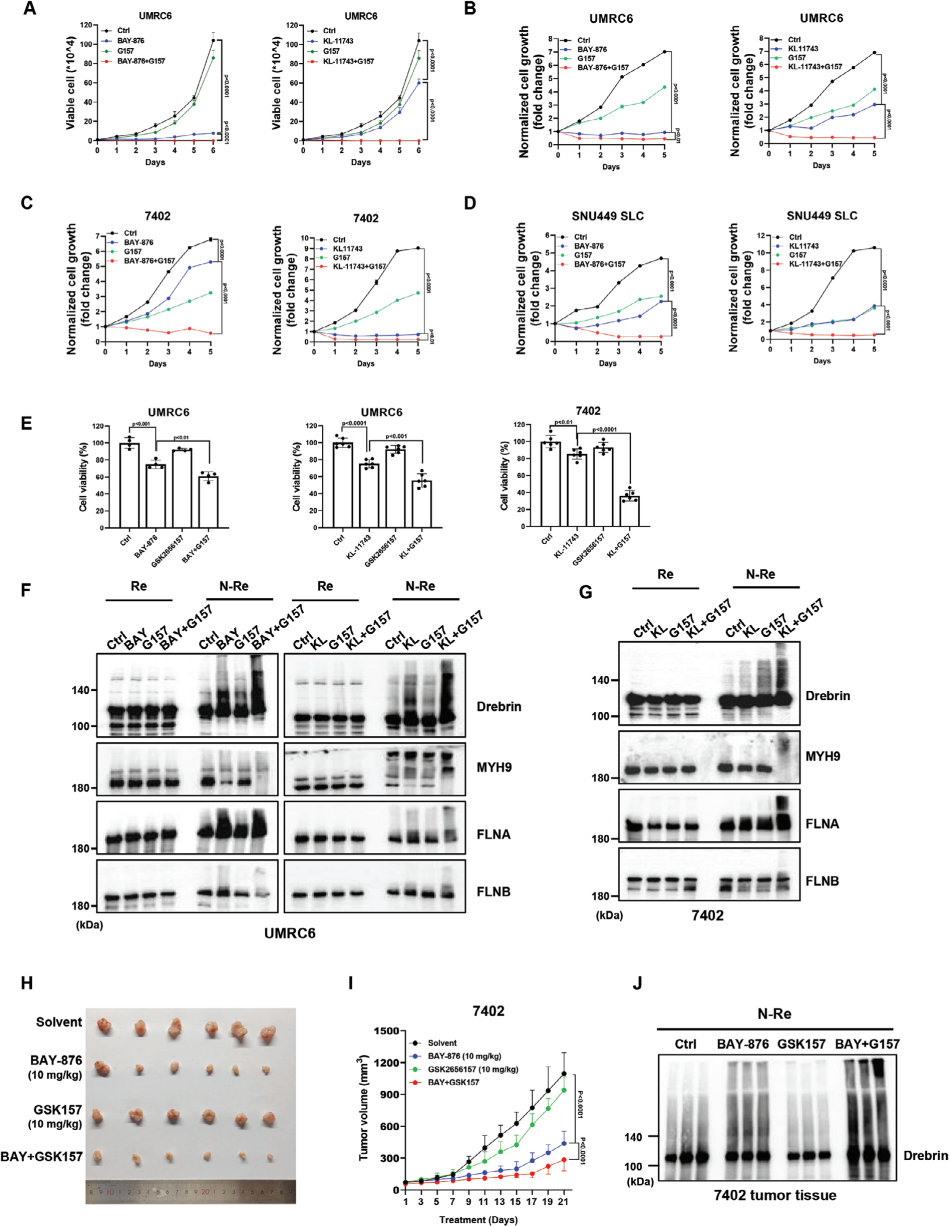
Inhibition of ER stress collaborates with GLUT inhibitors to promote disulfidptosis and suppress tumor growth. A) Viable cell count by Trypan blue staining of UMRC6 cells treated with or without BAY‐876 (0.5 µm), KL11743 (1 µm), and G157 (10 µm) for indicated treatment duration. B) Long‐term cell growth measurement by colony formation assay of UMRC6 (B), 7402 C) and SNU449‐SLC D) cells treated with or without BAY‐876, KL11743 and G157 for indicated treatment duration. E) Cell viability measured by CCK8 assay of UMRC6 and 7402 cells treated with or without BAY‐876 (5 µm), KL‐11743 (10 µm) and G157 (10 µm) for 4–6 h. F,G) Reducing and non‐reducing Western blotting analysis of cytoskeletal proteins in UMRC6 (F) and 7402 (G) cells treated with or without BAY‐876 (5 µm), KL‐11743 (10 µm) and G157 (10 µm) for 4–6 h. H) Photo of 7402 tumor tissues after treatment. Detailed treatment of in vivo experiments is described in the Materials and Methods. I) Tumor volumes of 7402 xenograft model with indicated treatments over time. J) Non‐reducing Western blotting analysis of Drebrin in 7402 xenograft tumor tissues with indicated treatments. All *p* values were calculated using a two‐tailed unpaired Student's *t*‐test. Data are mean ± SD, *n* ≥ 3 independent repeats. All Western blotting was repeated at least twice, independently, with similar results.

Our findings thus far indicate that inhibiting ER stress should promote disulfidptosis caused by glucose limitation or pharmacological interventions such as glucose transporter (GLUT) inhibition. BAY‐876 is a highly selective GLUT1 inhibitor with good metabolic stability in vitro and high oral bioavailability in vivo.^[^ ^32^ ^]^ KL‐11743 is a recently developed effective, orally active glucose‐competitive Class I GLUT inhibitor (GLUTi) with inhibitory activity on GLUT1 to GLUT4.^[^ ^33^ ^]^ Previous reports show that BAY‐876 or KL‐11743 could trigger disulfidptosis in SLC7A11‐high cells like glucose deprivation did.^[^ ^6^ ^]^ We showed that ER stress inhibitor G157 or GLUTi including BAY‐876 and KL‐11743 individually suppressed tumor growth to varied extents in SLC7A11‐high cells, while combined treatments of both almost completely inhibited tumor growth and even reduced the cell growth to a level lower than cells at the beginning of the treatments (**Figure** **6**A–D; Figure S6A,B, Supporting Information). In addition, we confirmed that G157 significantly sensitized tumor cells to GLUT inhibition mediated by BAY‐876 or KL‐11743 (Figure 6E), similar to its effect on cell survival under glucose deprivation conditions as shown in Figure 5A,B. Then, we determined the migration pattern of cytoskeletal proteins under non‐reducing conditions and found G157 strengthened the band retardation of cytoskeletal proteins including Drebrin, MYH9, FLNA, and FLNB, suggesting the induction of disulfide stress upon inhibition of ER stress and GLUTi together (Figure 6F,G). Consistent with our above results, GLUT inhibitors including BAY‐876 and KL‐11743 increased NADP^+^/NADPH ratio through blocking glucose/PPP pathway‐mediated NADPH generation, while the combination of G157 treatment decreased NADP^+^/NADPH ratio even though G157 promoted cell death caused by GLUT inhibitors (Figure S6C, Supporting Information). These results again suggest that G157 promoted disulfidptosis caused by GLUTi not through decreasing NADPH level or increasing NADP^+^/NADPH ratio. To explore the therapeutic application in vivo, we inoculated 7402 cells into nude mice to generate xenografts, and the results show that treatment with BAY‐876 decreased the growth of 7402 xenograft tumors, indicating that SLC7A11‐high tumor cells are sensitive to glucose inhibition. Additionally, though G157 alone had less effect on tumor growth, combined treatments with BAY‐876 and G157 further suppressed tumor growth in 7402 xenograft models (Figure 6H,I; Figure S6D,E, Supporting Information), consistent with the promotive effect of G157 on disulfidptosis in vitro. Next, we examined the band trailing of Drebrin of tumor tissues in different administration groups under non‐reducing conditions and found that the band trailing of Drebrin was stronger in the combined administration group than in the BAY‐876 treatment group (Figure 6J). Together, our data indicate that inhibition of ER stress response enhances GLUT inhibitors‐induced disulfidptosis and subsequent tumor suppression in SLC7A11‐high tumor cells.

## 3 Discussion

**Figure 7:**
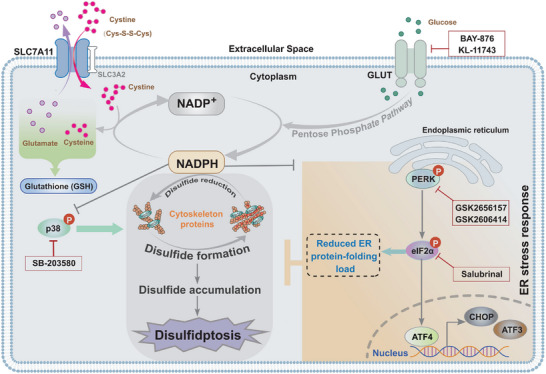
Proposed working model.

Unlike other types of regulated cell death, disulfidptosis is triggered by high cystine uptake coupled with NAPDH depletion under glucose starvation conditions, which leads to aberrant disulfide bonding in actin cytoskeletal proteins. The data presented in this study further explore key cellular circuits linked to disulfidptosis regulation and demonstrate that ER stress response is activated during disulfidptosis induced by SLC7A11 and glucose limitation. Pharmacological inhibition of ER stress response promotes disulfidptosis, while induction of ER stress suppresses disulfide stress‐caused cell death (**Figure** **7**). Besides, combined treatments with ER stress inhibitors and glucose transporter inhibitor exhibits great potency in suppressing tumor growth, highlighting the possibility of targeting disulfidptosis in tumor therapy.

Cellular signaling pathways not only participate in the functional activities during regulated cell death but also reflect specific reactions and might serve as a marker for particular events. Disulfidptosis is characterized by disulfide‐bond formation in actin cytoskeletal proteins, which must be monitored by non‐reducing western blotting. We employed an antibody array to identify p38 phosphorylation as an indicator for disulfidpotosis, since prevention of disulfide stress by inhibiting SLC7A11 or disulfide‐reducing agents completely blocks p38 phosphorylation (Figure 1I–M). The p38 mitogen‐activated protein kinase (MAPK) is an important mediator of signal transduction that responds to extracellular stressors such as UV radiation, hypoxia, pro‐inflammatory cytokines, and oxidative stress.^[^ ^34^ ^]^ In this study, we found that disulfidptosis was accompanied by an up‐regulation of total ROS levels but not mito‐ROS levels (Figure 2A–C; Figure S5C,D, Supporting Information). However, ROS scavenger TEMPOL or Trolox failed to abolish the phosphorylation of p38 during disulfidptosis, suggesting glucose starvation‐generated ROS is not the reason for p38 activation. Besides, previous findings connecting the ROS with the activation of the p38 pathway‐mediated apoptosis,^[^ ^35^ ^]^ whereas ROS scavengers couldn't suppress glucose deprivation‐induced disulfidptosis (Figure 2D–F), indicating the distinct functions of p38 activation in the two types of RCD. Several studies have suggested an association between disulfide stress and p38 activation. Ciriolo et al. reported that glutathione disulfide (GSSG) induced apoptosis by the activation of the p38 MAPK pathway, and blockage of p38 MAPK by specific inhibitor SB203580 alleviated cell death.^[^ ^36^ ^]^ Hydrogen peroxide (H_2_O_2_) is a well‐established activator of the p38 MAPK signaling pathway, however, the mechanisms of H_2_O_2_‐induced p38 activation are not yet fully understood.^[^ ^37^ ^]^ Because H_2_O_2_ is closely related to disulfide bond formation, the activation of the p38 pathway might involve the formation of disulfide bonds.^[^ ^38^ ^]^ p38 activation was observed in multiple studies reporting diallyl disulfide‐induced cell death, but the role of disulfide stress in cell death regulation is not fully explored.^[^ ^39^, ^40^ ^]^ Activation of p38 MAPK in response to various stress conditions has been shown to trigger host cell death through apoptosis or necroptosis,^[^ ^41^, ^42^, ^43^ ^]^ while our research further discovered the role of p38 in modulating disulfidptosis. Detailed mechanisms underlying the regulation of p38 by disulfide stress and how p38 activation promotes disulfidptosis remain for further investigation.

NADPH acts as an important cofactor of Trx‐disulfide reductases (TrxRs) and the glutathione disulfide (GSSG)‐reductases (Gsrs), which can reduce intracellular disulfides.^[^ ^44^ ^]^ Rapid depletion of NADPH in SLC7A11‐high cells upon glucose starvation is considered the major cause of disulfide bond formation during disulfidptosis. We found that inhibition of ER stress dramatically increased disulfide bonding in cytoskeletal proteins without further decreasing NADPH levels in the treated cells (Figure 5E), suggesting that ER stress may regulate disulfidptosis in an NADPH‐independent manner. The previous study of disulfidptosis conducted a whole‐genome CRISPR/Cas9 screening in SLC7A11‐overexpressing 786‐O cells under glucose‐deprivation conditions, which provides a bunch of candidate genes possibly involved in the regulation of disulfidptosis.^[^ ^6^ ^]^ Thus, to identify ER stress‐related genes that might regulate disulfidptosis, we conducted an analysis to identify the overlapping genes present in both the CRISPR/Cas9 screening results and the ER stress response gene list. The overlapping genes like OS9, RNF183, DERL2, ERLIN1, PDIA2, SEL1L, and TXNDC12 with a minus NormZ score, were considered as synergistic hits when knockout, meaning that activation of these genes should protect from glucose starvation‐induced cell death. Among them, OS9, DERL2, and SEL1L are responsible for ER quality control; RNF183 mediates prolonged ER stress‐triggered apoptosis; ERLIN1 regulates ER‐associated degradation; PDIA2 and TXNDC12 are members of PDI (Figure S7A,B, Supporting Information). ER‐localized PDI catalyzes the formation, breakage, and rearrangement of disulfide bonds, which is also induced during ER stress and responsible for balancing proteostasis.^[^ ^45^ ^]^ In addition, Tirosh et al. reported that when the ER stress response is activated, ER stress inhibitor GSK414 can induce the band trailing of ERp44 (a member of the PDI family), KIT, and cMET under non‐reducing conditions,^[^ ^46^ ^]^ which is consistent with our observation for the effects of GSK414 on disulfide bond accumulation. However, the regulation of cell death is not well studied in this report.^[^ ^46^ ^]^ In the future, it's worth figuring out the exact role of PDI as well as other ER‐related genes in the regulation of disulfidptosis, which would provide more insights into the mechanistic regulation of this unique form of cell death.

In summary, our study demonstrates the protective effect of the ER stress response on glucose limitation‐induced disulfidptosis in SLC7A11‐high tumor cells. Therapeutic inhibitors targeting ER stress response sensitize tumor cells to GLUT inhibitors‐induced disulfidptosis and dramatically suppress tumor growth, conceptualizing combined therapy by targeting disulfidptosis in cancer treatments.

## 4 Experimental Section

### Cell Culture Studies

HEK293T (CRL‐3216), 786‐O (CRL‐1932), H460 (HTB‐177), and SNU449 (CRL‐2234) cell lines were obtained from ATCC. The UMRC6 cell line was originally purchased from Sigma (#0 809 0513, 2017). BEL‐7402 was provided by Dr. Kangsheng Tu at The First Affiliated Hospital of Xi'an Jiaotong University, and the RCC4 cell line was a gift from Dr. Boyi Gan at The University of Texas MD Anderson Cancer Center, Houston. The above cell lines were maintained and frozen in the laboratory and were tested to be Mycoplasma‐free annually. All cell lines were cultured in DMEM supplemented with 10% FBS and 1% penicillin‐streptomycin antibiotics except for the H460 cell line, which was cultured in the RPMI‐1640 medium. A special DMEM medium (lack of glucose and cystine) was customized by Quanxin Biotechnology (China). For glucose or cystine deprivation experiments, a certain amount of cystine or glucose was re‐added to prepare the corresponding medium.

### Constructs and Stable cell Line Generation

Gene knockout was performed by sgRNAs and CRISPR/Cas9 technology. CRISPR‐mediated knockout plasmids containing guide RNAs targeting SLC7A11 and MAPKs (Table S1, Supporting Information) were generated in LentiCRISPR v2 (Addgene, #52 961) according to the protocol. Human SLC7A11, SLC7A11^C86S mutant^, and SOD2 cDNAs were cloned into the lentivirus vector pLVX‐puro with an N‐terminal FLAG tag. All constructs were confirmed by DNA sequencing. Stable cell lines were generated as follows: HEK293T cells were transfected with lentiviral constructs, together with psPAX.2 and pMD2.G third‐generation lentiviral packaging system using Lipofectamine 2000 reagent (Life Technologies) as the manufacturer's instructions. The lentivirus particles were collected and the target cell lines were then infected with polybrene transfection reagent (Solarbio, H8761, China). Stable cell lines were screened with puromycin (Solarbio, P8230) after virus infection.

### Reagents and Antibodies

The reagents were purchased as followings: Z‐VAD‐FMK (T7020), Necrostain 2 racemate (S8641), Rotenone (T2970) and MitoQ10 (T28049) were bought from TargetMol (China); GSH‐MEE (G1404), tert‐Butyl hydroperoxide (458 139), D‐Penicillamine (P4875) and L‐Penicillamine (196 312) were bought from Sigma‐Aldrich (USA); Antimycin A1 (C4452) was from APExBIO (USA); NADP^+^/NADPH detection kit (S0179) was from Beyotime (China); D‐Glucose (A501991), L‐cystine (A610088), propidium iodide (A601112), 2‐Deoxy‐D‐glucose (A602241), N‐Acetyl‐L‐cysteine (A601127) were obtained from Sangon Biotech (China); RSL3 (S8155), Chloroquine (S6999), Erastin (S7242) and Liproxstain‐1 (S7699) were from Selleck (USA); Staurosporine (HY‐15141), Sulfasalazine (HY‐14655), SB203580 (HY‐10256), BSO (HY‐106376), Metformin (HY‐B0627), Phenformin (HY‐16397A), Brefeldin A (HY16592), Thapsigargin (HY‐13433), Tunicamycin (HY‐A0098), GSK2656157 (HY‐13820), GSK2606414 (HY‐18072), Salubrinal (HY‐15486), Trolox (HY‐101445), BAY‐876 (HY‐100017), KL‐11743 (HY‐145597) were purchased from MedChemExpress (USA). All reagents were dissolved according to manufacturers’ instructions.

The primary antibodies used for Western blotting assays were as followings: Drebrin (10260‐1‐AP), β‐Tubulin (66240‐1‐Ig) and Vinculin (66305‐1‐Ig) were bought from Proteintech (China); β‐actin (AC004), FLNA (A3738), FLNB (A2481), MYH9 (A0173), TLN1 (A4158) were obtained from Abclonal Technology (China); SLC7A11 (#12 691), p38 (#8690), P‐p38 (#4511), cleaved Caspase‐3 (#9661), PARP (#9532), LC3B (#3868), P‐JNK (#4668), XBP‐1s (#12 782), NRF2 (#12 721), eIF2α (#5324), P‐eIF2α (#3398), ATF3 (#33 593) and ATF4 (#11 815) were obtained from Cell Signaling Technology (USA). MAPK12 (#HA722017) and MAPK14 (#ET1602‐26) were obtained from HUABIO (China).

### Cell Viability and Death Assays

Cell viability was determined by Cell Counting Kit‐8 reagent (CCK8, USA) or crystal violet staining. For CCK8 assays, cells were planted in 96‐well plates and were treated the next day. Then, the medium in each well was replaced with fresh medium containing CCK‐8 reagent. After 1‐h incubation, the plates were read by a microplate reader (Thermo Scientific, USA) at an absorbance of 450 nm. For crystal violet staining assays, cells were seeded in 12‐well plates and treated, followed by fixing with methanol for 20 min and staining with 0.1% crystal violet solution for 20 min. Then, the plates were washed with water until the bottom became clean, and then the plates were dried and 1% SDS was added to dissolve the crystal violet. The equal‐volume supernatant was then transferred to a 96‐well plate and read by a microplate reader at an absorbance of 570 nm. Cell death was measured by PI staining. In brief, cells were seeded in 12‐well plates a day before treatment. After treatment with the designed culture medium or drug, cells were trypsinized and collected in an EP tube, washed with PBS, and stained with 2 µg mL^−1^ PI in PBS. Dead cells (PI‐positive) were recognized with a flow cytometer (BD Biosciences, USA), analyzed, and plotted by FlowJo 10 software. In the viable cell count assay, cells were collected at different time points and stained with Trypan blue (Solarbio, T8070), and then live cells were counted by a cell counter (Luna‐II, China).

### Western Blotting

Briefly, cell lysates were harvested by NP‐40 buffer (150 mm sodium chloride, 1.0% NP‐40, 50 mm Tris, pH 8.0), followed by ultrasound, centrifugation, supernatant collection, protein concentration determination, and protein denaturing. The same amount of proteins was loaded and separated via an SDS‐PAGE gel and transferred to a PVDF membrane. The membrane was then blocked in 5% skim milk, followed by incubation in primary and secondary antibodies before visualization by an enhanced chemiluminescence system (Baygene Biotech, China). For non‐reducing Western blotting assays, protein samples were split into two aliquots after concentration determination. One of the aliquots was denatured with a loading buffer without any reducing agent. Both of the two aliquots were incubated at 70 °C for 10 min.

### Real‐Time PCR

The total mRNA of each sample was extracted by using Trizol reagent (ABclonal, RK30129). 2 µg of RNA was subjected to reverse transcription of cDNA by using ABScript III RT Master Mix for qPCR (ABclonal, RK21428). Real‐time PCR was performed in a 20 µL reaction mixture system by using 2× Universal SYBR Green Fast qPCR Mix (ABclonal, RK21203). GAPDH was used as internal control. The primer sequences used are listed in Table S2 (Supporting Information).

### Cystine Uptake Assay

Cells were seeded in 12‐well plates, and each well was replaced with cystine uptake medium (containing 1 µm cystine and 0.04 µCi 2‐[1‐14 C] labeled cystine, PekinElmer, USA) after cells attachment. Next, the plates were incubated and followed by washing with PBS and lysing in 0.1 mm NaOH. Radioactivity of intracellular labeled cystine was measured with a Tri‐Carb Liquid Scintillation Analyzer (PerKinElmer) according to the manufacturer's instructions.

### Reactive Oxygen Species (ROS) Level and Mitochondrial ROS Measurement

Cells were seeded in 12‐well plates and treated. After that, cells were stained with fresh medium containing 10 µm H2DCFDA (Thermo Fisher Scientific, D399) or 5 µm MitoSOX (Thermo Fisher Scientific, M36007) for 30 min, washed with PBS and subjected to flow cytometry (FACS) analysis by a flow cytometer (BD Biosciences), and plotted using FlowJo 10 software.

### GSH Measurement

The GSH and GSSG Assay Kit (S0053) was purchased from Beyotime Biotechnology (China) for GSH measurement. The principle of this experiment is as follows: glutathione consists of reduced glutathione (GSH) and oxidized glutathione disulfide (GSSG). GSH can react with DTNB to produce TNB (yellow) and GSSG. When GSSG is reduced to GSH with glutathione reductase, the total glutathione content can be measured based on the TNB absorbance. After removing GSH from the sample, the GSSG content can be measured by the above method, and the difference between total glutathione and GSSG is the amount of GSH. This assay was conducted according to the manufacturer's instructions.

### NADP^+^/NADPH Measurement

NADP^+^/NADPH Assays were performed using a kit (S0179) purchased from Beyotime Biotechnology and according to the manufacturer's instructions. In short, cells were treated and an extraction buffer was added to collect the lysate. The collected supernatants were then centrifuged and divided into two aliquots, one of which was heated at 60 °C for 30 min to remove NADP^+^. Both the heated and unheated samples were then transferred to a 96‐well plate and a G6PDH working solution and chromogenic agent were added. G6PDH can reduce NADP^+^ to NADPH, and NADPH can reduce WST‐8 to formazan. The amount of NADPH can therefore be calculated based on the formazan produced. The 96‐well plate was then measured at 450 nm by a microplate reader.

### Gene Expression Correlation in TCGA Cancers

The expression data of ATF3 and glucose metabolism genes were obtained from the Cancer Genome Atlas (TCGA) database. Pearson's correlation (two‐sided) analysis was performed using R software to map the expression correlation between ATF3 and glucose metabolism genes.

### Xenograft Experiments

The xenograft experiments were performed in accordance with the National Guidelines for Experimental Animal Welfare and with approval of the Ethics Committee of Xi'an Jiaotong University. 4 to 6‐week‐old female balb/c nude mice were purchased from Beijing Vital River Laboratory Animal Technology Co, Ltd., and were used for cell line xenograft experiments. The 7402 cells were resuspended in the FBS‐free DMEM medium and were injected into mice subcutaneously. The tumor volume was calculated according to the equation: volume = 0.5 × length × width^2^. When the average tumor volume had reached ≈50–100 mm^3^, the mice were assigned randomly into 4 groups, and were treated with the solvent, 10 mg kg^−1^ BAY‐876, 10 mg kg^−1^ GSK2656157, or 10 mg kg^−1^ BAY‐876 + 10 mg kg^−1^ GSK2656157 through intraperitoneal injections every two days. Animals were sacrificed when the average tumor volume reached 1000 mm^3^, and the treatment was terminated after 3 weeks.

### Statistical Analysis

Statistical analyses and graph plotting in this study were conducted by GraphPad Prism software. All *p* values were calculated using two‐tailed unpaired Student's *t*‐test. Data were presented as mean ± SD. Statistical significance was determined if *p* < 0.05. ns refers to not significant (*p* > 0.05). All cell culture experiments were repeated at least three times, and all Western blotting was repeated at least twice, independently, with similar results.

### Ethics Approval

All animal experiments were performed under the National Guidelines for Experimental Animal Welfare and with the approval of the Ethics Committee of Xi'an Jiaotong University. The approval number of the xenograft experiment in this study is XJTUAE2023‐1500.

## Conflict of Interest

The authors declare no conflict of interest.

## Supporting information



Supporting Information

## Data Availability

The data that support the findings of this study are available from the corresponding author upon reasonable request.
