# Supplementary material for: Inhibition of Endoplasmic Reticulum Stress Cooperates with SLC7A11 to Promote Disulfidptosis and Suppress Tumor Growth upon Glucose Limitation
Source: Adv Sci (Weinh). 2024 Dec 30;12(7):2408789. doi: 10.1002/advs.202408789 (PMC11831432; doi:10.1002/advs.202408789)
Supplement: Supplementary file 1 — Supporting Information [file ADVS-12-2408789-s001.pdf]

ADVANCED SCIENCE
Open Access

Supporting Information

for *Adv. Sci.*, DOI 10.1002/advs.202408789

Inhibition of Endoplasmic Reticulum Stress Cooperates with SLC7A11 to Promote Disulfidptosis and Suppress Tumor Growth upon Glucose Limitation

Jin Wang, Jing Chen, Kexin Fan, Minglin Wang, Min Gao, Yakun Ren, Shaobo Wu, Qian He, Kangsheng Tu, Qiuran Xu* and Yilei Zhang**

Fig.S1

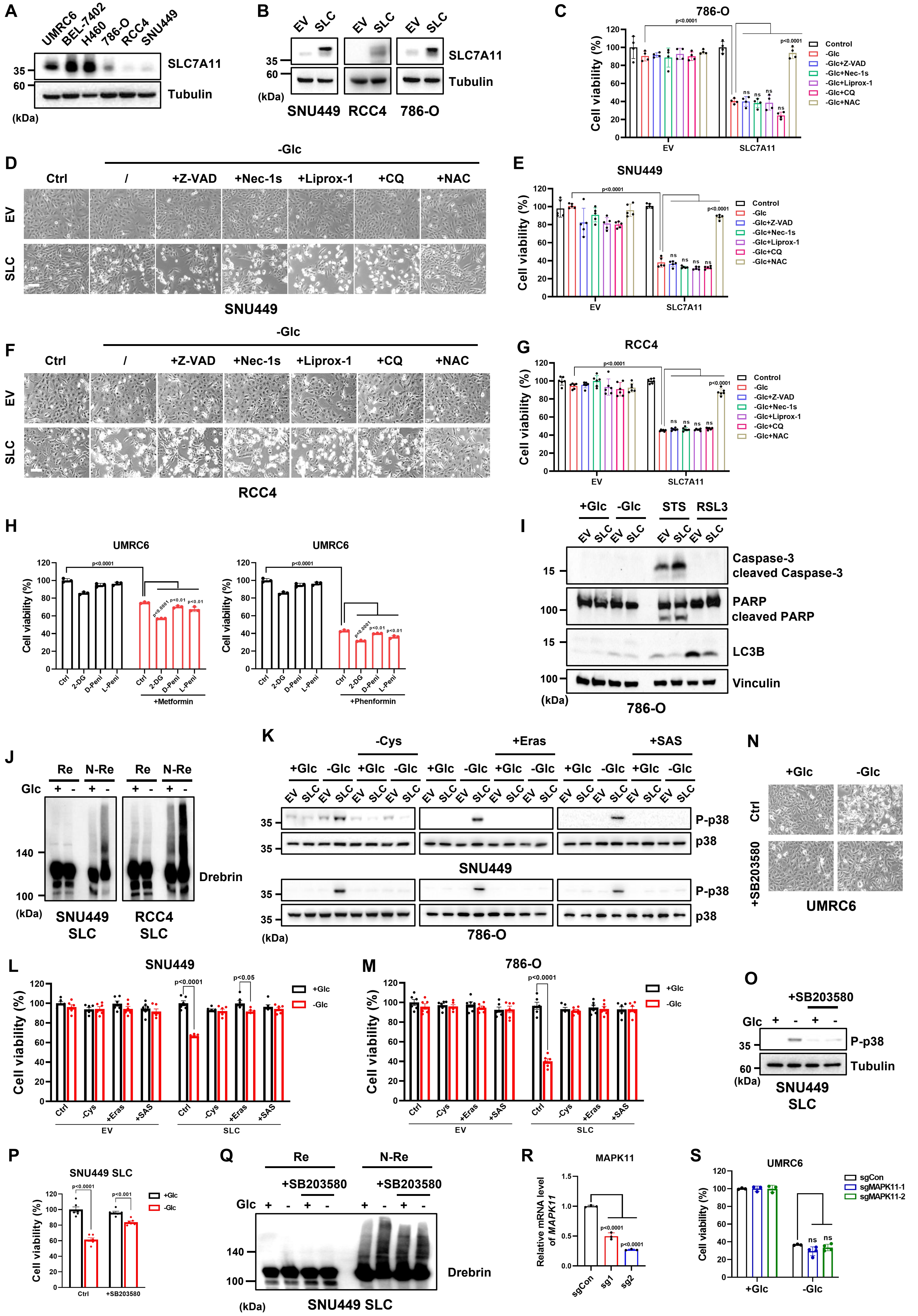
A
UMRC6
BEL-7402
H460
786-O
RCC4
SNU449
SLC7A11
Tubulin
(kDa)
B
EV
SLC
SNU449
RCC4
786-O
C
786-O
Cell viability (%)
Control
-Glc
-Glc+Z-VAD
-Glc+Nec-1s
-Glc+Liprox-1
-Glc+CQ
-Glc+NAC
EV
SLC7A11
D
-Glc
Ctrl
/
+Z-VAD
+Nec-1s
+Liprox-1
+CQ
+NAC
SNU449
E
SNU449
F
RCC4
G
RCC4
H
UMRC6
2-DG
D-Peni
L-Peni
+Metformin
+Phenformin
I
+Glc
STS
RSL3
Caspase-3
cleaved Caspase-3
PARP
cleaved PARP
LC3B
Vinculin
786-O
J
Re
N-Re
Glc
Drebrin
SNU449 SLC
RCC4 SLC
K
-Cys
+Eras
+SAS
P-p38
p38
N
UMRC6
+SB203580
L
M
O
P
SNU449 SLC
Q
R
MAPK11
Relative mRNA level of MAPK11
sgCon
sg1
sg2
S
sgMAPK11-1
sgMAPK11-2

Fig S1. SLC7A11 promotes p38 phosphorylation during disulfidptosis induced by glucose deprivation

A. Western blotting analysis of SLC7A11 expression in the indicated cell lines used in this study. **B**. Western blotting analysis of SLC7A11 expression in empty vector (EV) and SLC7A11 overexpressing SNU449, RCC4 and 786-O cell lines. **C-G**. Cell morphological changes (**D&F**) and cell viability (**C&E&G**) measured by CCK8 assay in EV and SLC7A11 overexpressing SNU449, RCC4 and 786-O cells cultured in glucose-containing (Ctrl) or glucose-free (-Glc) medium with or without Z-VAD (5 μM), Nec-1s (2 μM), Liprox-1 (5 μM), CQ (20 μM) and NAC (2 mM) for 4-6 h. Scale bars, 100 μm. **H**. Cell viability measured by CCK8 assay in UMRC6 cells treated with energy stress inducers 2-DG (10 mM), D-/L-penicillamine (1 mM) with or without metformin (2 mM) and phenformin (2 mM). **I**. Western blotting analysis of apoptotic markers in EV and SLC7A11 overexpressing 786-O cells cultured in glucose-containing/-free medium with or without Staurosporine (STS, 1 μM) and RSL3 (10 μM). **J**. Reducing and non-reducing Western blotting analysis of Drebrin in SNU449-SLC and RCC4-SLC cells cultured in glucose-containing/-free medium for 6-8 h. **K**. Western blotting analysis of p38 phosphoryation in SNU449 and 786-O cells cultured in glucose-containing/-free medium with or without Cystine, Erastin (10 μM) and Sulfasalazine (SAS, 10 μM). **L-M**. Cell viability measured by CCK8 assay in SNU449 (**L**) and 786-O (**M**) cells cultured in glucose-containing/-free medium with or without indicated concentrations of Cystine, Erastin and SAS. **N**. Cell morphological changes in UMRC6 cells cultured in glucose-containing/-free medium with or without SB203580 (50 μM) treatment. Scale bars, 100 μm. **O**. Western blotting analysis of p38 phosphoryation in SNU449-SLC cells cultured in glucose-containing/-free medium with or without SB203580. **P**. Cell viability measured by CCK8 assay in SNU449-SLC cells cultured in glucose-containing/-free medium with or without SB203580. **Q**. Reducing and non-reducing Western blotting analysis of Drebrin in SNU449-SLC cells cultured in glucose-containing/-free medium with or without SB203580. **R**. RT-PCR analysis of MAPK11 levels in control and MAPK11 knockout UMRC6 cell lines. **S**. Cell viability measured by CCK8 assay in control and MAPK11 knockout UMRC6 cell lines cultured in glucose-containing/-free medium. All *P* values were calculated using two-tailed unpaired Student's *t*-test. Data are mean ± SD, n ≥ 3 independent repeats unless specified. ns: not significant ($P > 0.05$). All Western blotting was repeated at least twice, independently, with similar results.

Fig.S2

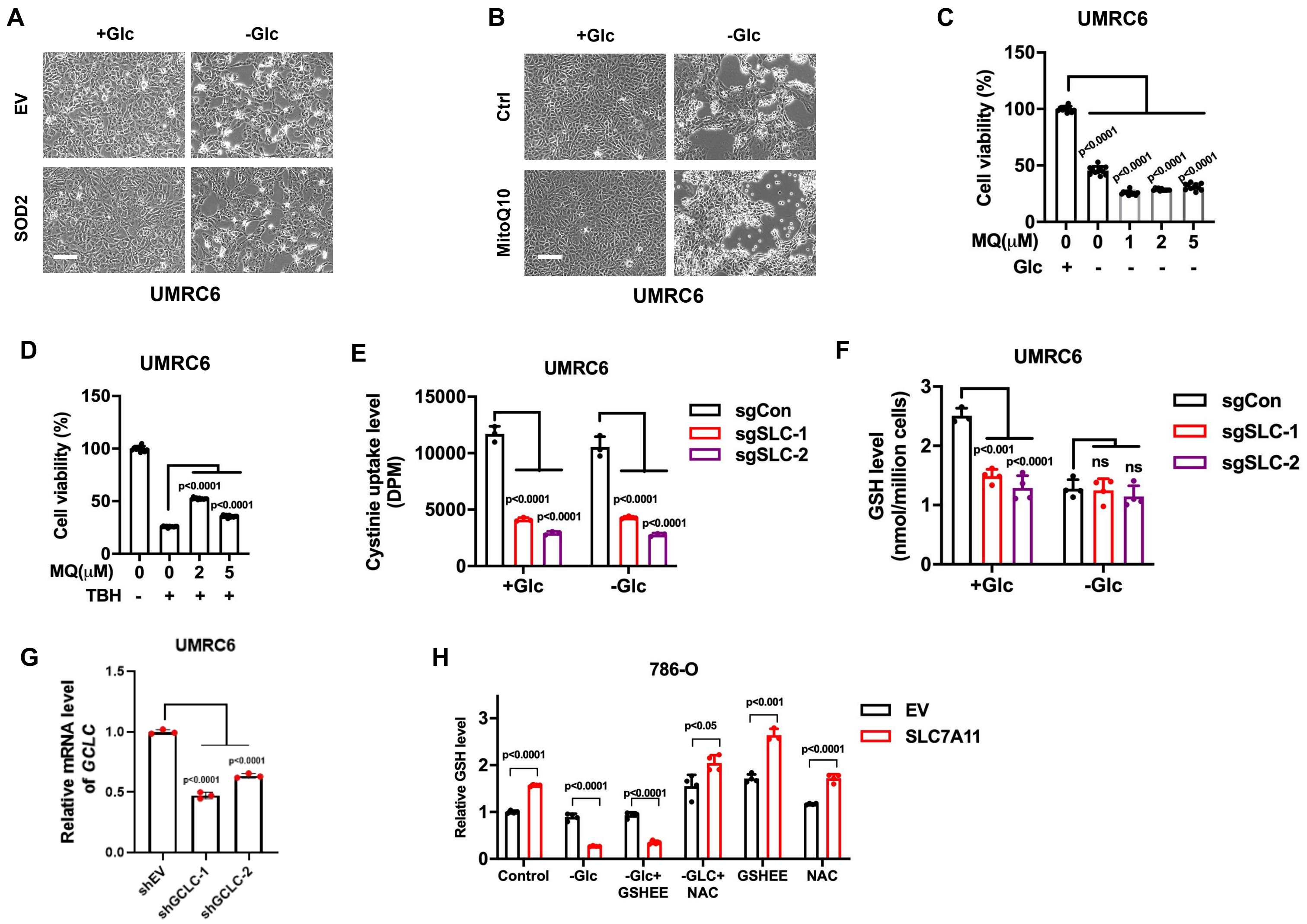
A
+Glc
-Glc
EV
SOD2
UMRC6
B
+Glc
-Glc
Ctrl
MitoQ10
UMRC6
C
UMRC6
Cell viability (%)
150
100
50
0
p<0.0001
p<0.0001
p<0.0001
p<0.0001
MQ(μM) 0 0 1 2 5
Glc + - - - -
D
UMRC6
Cell viability (%)
150
100
50
0
p<0.0001
p<0.0001
MQ(μM) 0 0 2 5
TBH - + + +
E
UMRC6
Cystinie uptake level (DPM)
15000
10000
5000
0
p<0.0001
p<0.0001
p<0.0001
p<0.0001
+Glc
-Glc
sgCon
sgSLC-1
sgSLC-2
F
UMRC6
GSH level (nmol/million cells)
3
2
1
0
p<0.001
p<0.0001
ns
ns
+Glc
-Glc
sgCon
sgSLC-1
sgSLC-2
G
UMRC6
Relative mRNA level of GCLC
1.5
1.0
0.5
0.0
p<0.0001
p<0.0001
shEV
shGCLC-1
shGCLC-2
H
786-O
Relative GSH level
3
2
1
0
p<0.0001
p<0.0001
p<0.0001
p<0.05
p<0.001
p<0.0001
Control
-Glc
-Glc+ GSHEE
-GLC+ NAC
GSHEE
NAC
EV
SLC7A11

Fig S2. Glucose deprivation induces disulfidptosis independent of mito-ROS and GSH

A-B. Cell morphological changes in UMRC6 cells cultured in glucose-containing/-free medium with or without SOD2 overexpression (**A**) or MitoQ10 (5 μM) treatment (**B**). Scale bars, 100 μm. **C**. Cell viability measured by CCK8 assay in UMRC6 cells cultured in glucose-containing/-free medium with or without indicated concentrations of MitoQ10. **D.** Cell viability measured by CCK8 assay in UMRC6 cells treated with or without indicated concentrations of MitoQ10 and TBH. **E-F**. Cystine uptake levels (**E**) and GSH levels (**F**) of control and SLC7A11 knockout UMRC6 cells cultured in glucose-containing/-free medium. **G**. RT-PCR analysis of GCLC levels in control and GCLC knockdown UMRC6 cell lines. **H**. Relative GSH levels of EV and SLC7A11 overexpressing 786-O cells cultured in glucose-containing/-free medium with or without GSHEE (200 μM) and NAC. All *P* values were calculated using two-tailed unpaired Student's *t*-test. Data are mean ± SD, n ≥ 3 independent repeats. ns: not significant (*P* > 0.05).

Fig.S3

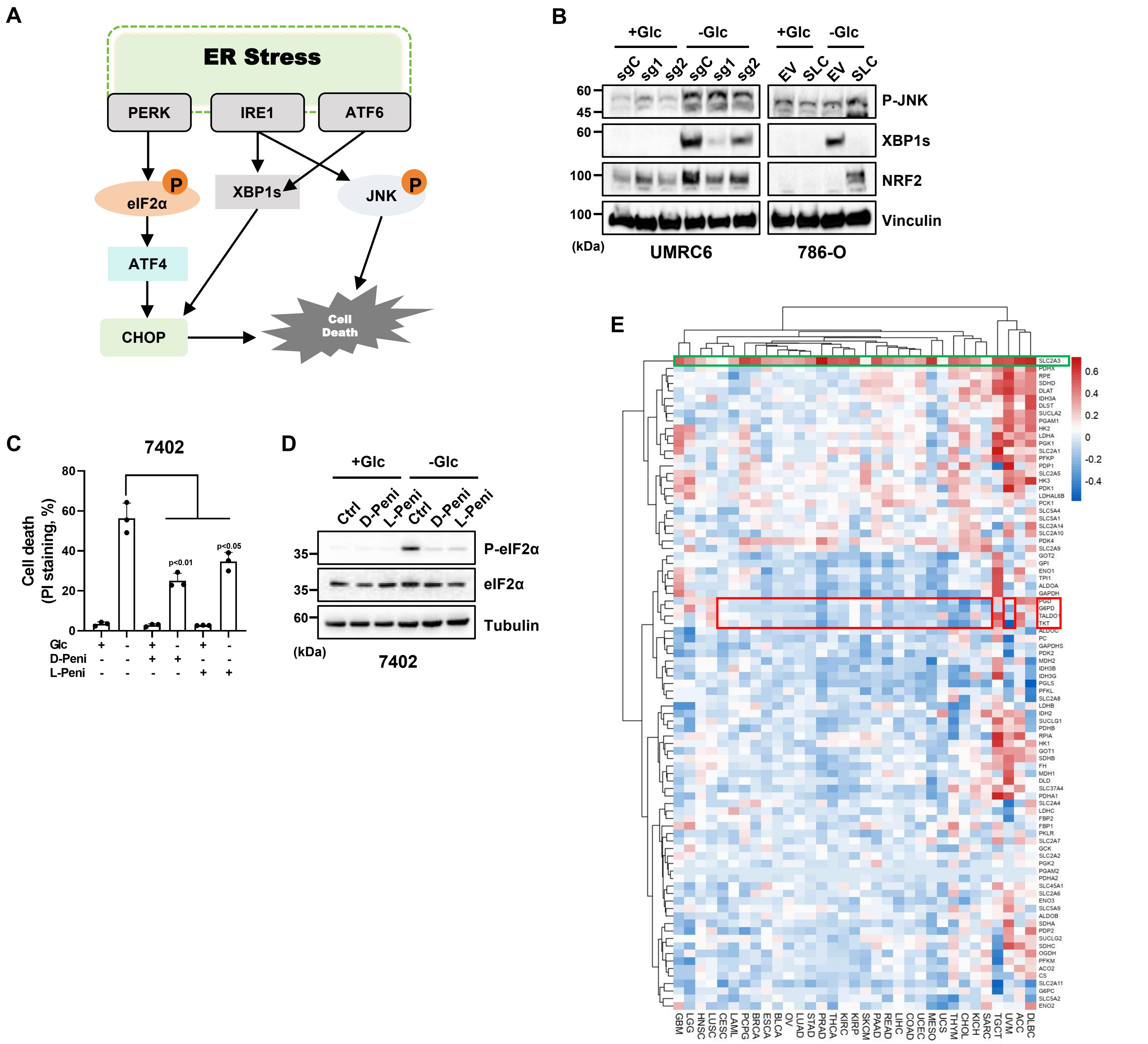
A
ER Stress
PERK
IRE1
ATF6
P
eIF2α
XBP1s
JNK
P
ATF4
CHOP
Cell Death
B
+Glc
-Glc
+Glc
-Glc
sgC sg1 sg2 sgC sg1 sg2
EV SLC EV SLC
60
45
60
100
100
P-JNK
XBP1s
NRF2
Vinculin
(kDa)
UMRC6
786-O
C
7402
Cell death (PI staining, %)
80
60
40
20
0
p<0.01
p<0.05
Glc + - + - + -
D-Peni - - + + - -
L-Peni - - - - + +
D
+Glc
-Glc
Ctrl D-Peni L-Peni Ctrl D-Peni L-Peni
35
35
60
P-eIF2α
eIF2α
Tubulin
(kDa)
7402
E
0.6
0.4
0.2
0
-0.2
-0.4
GBM LGG HNSC LUSC CESC LAML PCPG BRCA ESCA BLCA OV LUAD STAD PRAD THCA KIRC KIRP SKCM PAAD READ LIHC COAD UCEC MESO UCS THYM CHOL KICH SARC TGCT UVM ACC DLBC

Fig S3. ER stress response is activated during disulfidptosis caused by SLC7A11 and glucose deprivation

A. ER stress response downstreaming pathways. **B**. Western blotting analysis of P-JNK, XBP1s and NRF2 expressions in UMRC6 and 786-O cells cultured in glucose-containing/-free medium. **C**. Cell death measured by PI staining in 7402 cells cultured in glucose-containing/-free medium with or without D-/L-penicillamine. **D**. Western blotting analysis of eIF2α phosphorylation of 7402 cells cultured in glucose-containing/-free medium with or without D-/L-penicillamine. **E**. Correlation analysis on the expression of ATF3 and glucose metabolism genes in patients with tumors (data from TCGA database). All *P* values were calculated using two-tailed unpaired Student's *t*-test. Data are mean ± SD, n ≥ 3 independent repeats. All Western blotting was repeated at least twice, independently, with similar results.

Fig.S4

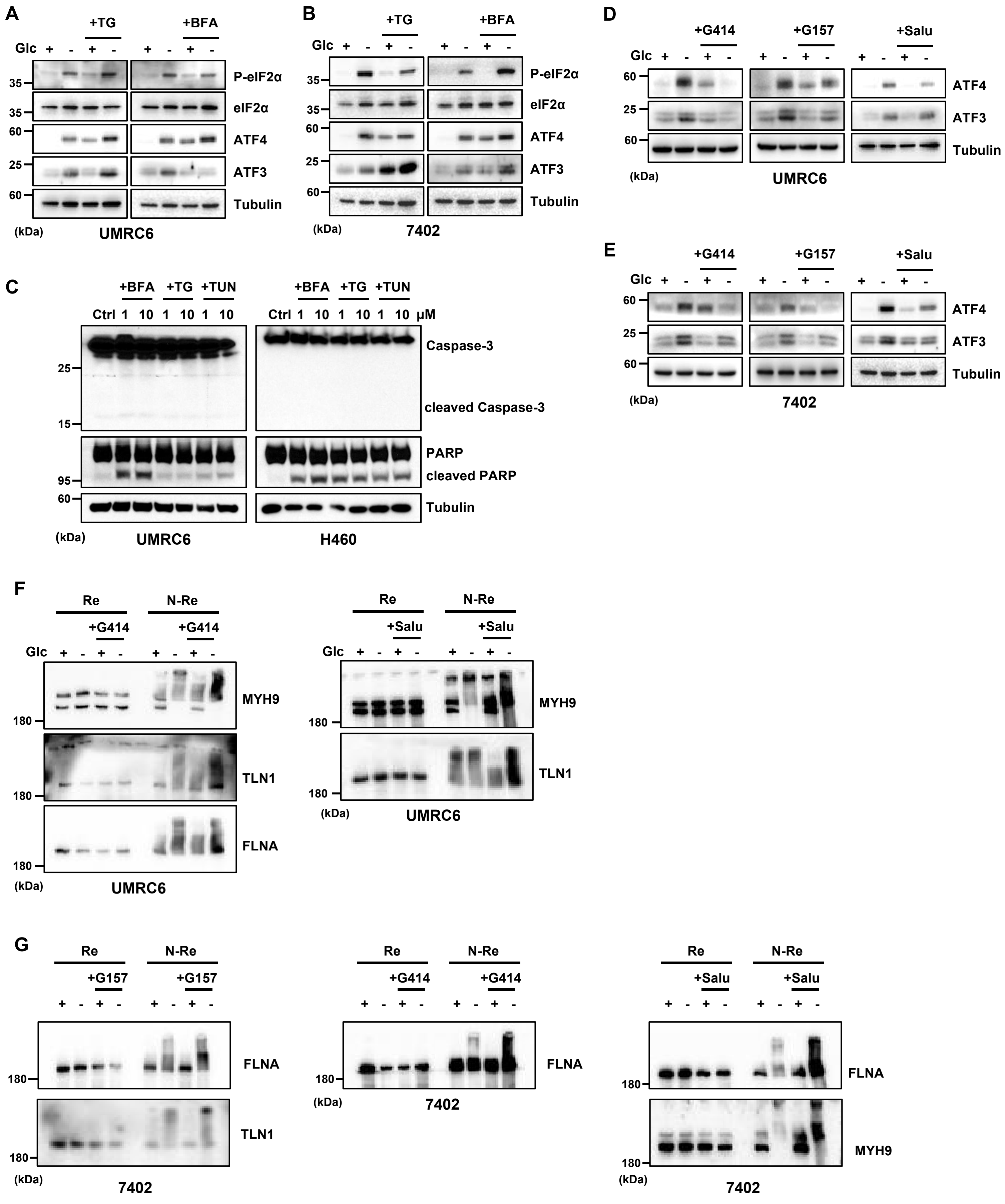
A
+TG
+BFA
Glc + - + - + - + -
35
35
60
25
60
P-eIF2α
eIF2α
ATF4
ATF3
Tubulin
(kDa)
UMRC6
B
+TG
+BFA
Glc + - + - + - + -
35
35
60
25
60
P-eIF2α
eIF2α
ATF4
ATF3
Tubulin
(kDa)
7402
D
+G414
+G157
+Salu
Glc + - + - + - + - + - + -
60
25
60
ATF4
ATF3
Tubulin
(kDa)
UMRC6
E
+G414
+G157
+Salu
Glc + - + - + - + - + - + -
60
25
60
ATF4
ATF3
Tubulin
(kDa)
7402
C
+BFA +TG +TUN
Ctrl 1 10 1 10 1 10
+BFA +TG +TUN
Ctrl 1 10 1 10 1 10 μM
25
15
95
60
Caspase-3
cleaved Caspase-3
PARP
cleaved PARP
Tubulin
(kDa)
UMRC6
H460
F
Re
N-Re
+G414
+G414
Glc + - + - + - + -
180
180
180
MYH9
TLN1
FLNA
(kDa)
UMRC6
Re
N-Re
+Salu
+Salu
Glc + - + - + - + -
180
180
MYH9
TLN1
(kDa)
UMRC6
G
Re
N-Re
+G157
+G157
+ - + - + - + -
180
180
FLNA
TLN1
(kDa)
7402
Re
N-Re
+G414
+G414
+ - + - + - + -
180
FLNA
(kDa)
7402
Re
N-Re
+Salu
+Salu
+ - + - + - + -
180
180
FLNA
MYH9
(kDa)
7402

Fig S4. Inhibition of ER stress response promotes disulfidptosis

A-B. Western blotting analysis of PERK/eIF2α/ATF4 pathway proteins in UMRC6 (**A**) and 7402 (**B**) cells cultured in glucose-containing/-free medium with or without TG (2 μM) and BFA (5 μM). **C**. Western blotting analysis of apoptotic markers in UMRC6 and H460 cells treated with indicated concentrations of TG, BFA and Tunicamycin (TUN, 5 μM). **D-E**. Western blotting analysis of ATF4 and ATF3 expressions UMRC6 (**D**) and 7402 (**E**) cells cultured in glucose-containing/-free medium with or without G414 (10 μM), G157 (10 μM) and Salu (20 μM). **F-G**. Reducing and non-reducing Western blotting analysis of cytoskeletal proteins in UMRC6 (**F**) and 7402 (**G**) cells cultured in glucose-containing/-free medium with or without G157, G414 and Salu. All Western blotting was repeated at least twice, independently, with similar results.

Fig.S5

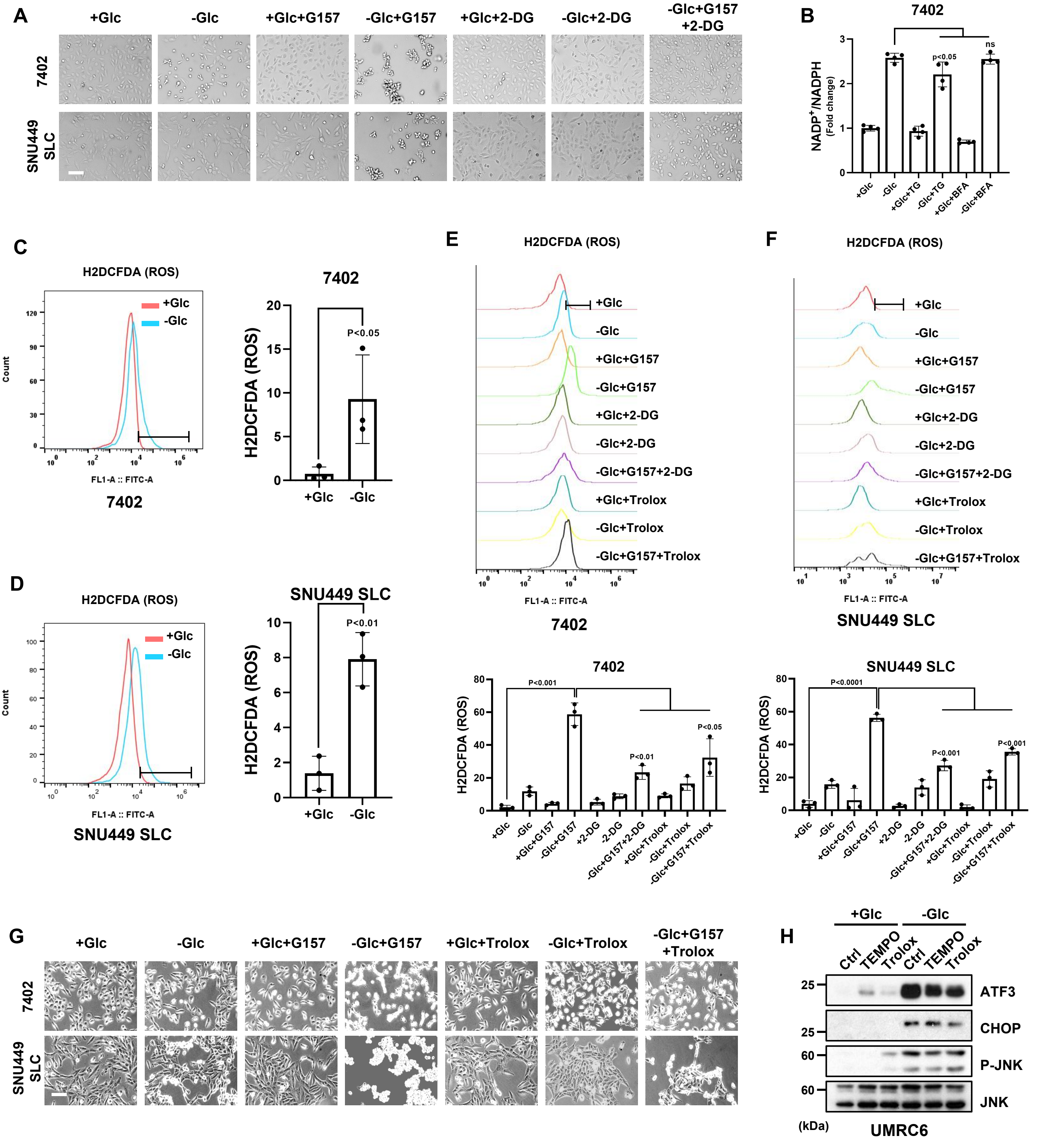
A
+Glc
-Glc
+Glc+G157
-Glc+G157
+Glc+2-DG
-Glc+2-DG
-Glc+G157 +2-DG
7402
SNU449 SLC
B
7402
NADP+/NADPH (Fold change)
p<0.05
ns
+Glc
-Glc
+Glc+TG
-Glc+TG
+Glc+BFA
-Glc+BFA
C
H2DCFDA (ROS)
+Glc
-Glc
Count
FL1-A :: FITC-A
7402
P<0.05
H2DCFDA (ROS)
D
H2DCFDA (ROS)
SNU449 SLC
P<0.01
E
H2DCFDA (ROS)
+Glc
-Glc
+Glc+G157
-Glc+G157
+Glc+2-DG
-Glc+2-DG
-Glc+G157+2-DG
+Glc+Trolox
-Glc+Trolox
-Glc+G157+Trolox
FL1-A :: FITC-A
7402
P<0.001
P<0.01
P<0.05
+2-DG
-2-DG
F
H2DCFDA (ROS)
SNU449 SLC
P<0.0001
P<0.001
P<0.001
G
+Glc
-Glc
+Glc+G157
-Glc+G157
+Glc+Trolox
-Glc+Trolox
-Glc+G157 +Trolox
7402
SNU449 SLC
H
+Glc
-Glc
Ctrl
TEMPO
Trolox
ATF3
CHOP
P-JNK
JNK
(kDa)
UMRC6

Fig S5. ER stress response suppresses disulfidptosis in a NADPH and ROS-independent manner

A. Cell morphological changes of 7402 and SNU449-SLC cells cultured in glucose-containing/-free medium with or without G157 (10 μM) and 2-DG (10 mM). Scale bars, 100 μm. **B**. $NADP^+$/NADPH ratios of 7402 cells cultured in glucose-containing/-free medium with or without TG and BFA. **C-D**. ROS levels measured by flow cytometry in 7402 (**C**) and SNU449-SLC (**D**) cells cultured in glucose-containing/-free medium. **E-F**. ROS levels measured by flow cytometry in 7402 (**E**) and SNU449-SLC (**F**) cells cultured in glucose-containing/-free medium with or without G157, Trolox (2 mM) and 2-DG. **G**. Cell morphological changes of 7402 and SNU449-SLC cells cultured in glucose-containing/-free medium with or without Trolox and 2-DG. Scale bars, 100 μm. **H**. Western blotting analysis of ATF3, CHOP and P-JNK levels in UMRC6 cells cultured in glucose-containing/-free medium with or without TEMPO (2 mM) and Trolox. All *P* values were calculated using two-tailed unpaired Student's *t*-test. Data are mean ± SD, n ≥ 3 independent repeats. ns: not significant ($P > 0.05$). All Western blotting was repeated at least twice, independently, with similar results.

Fig.S6

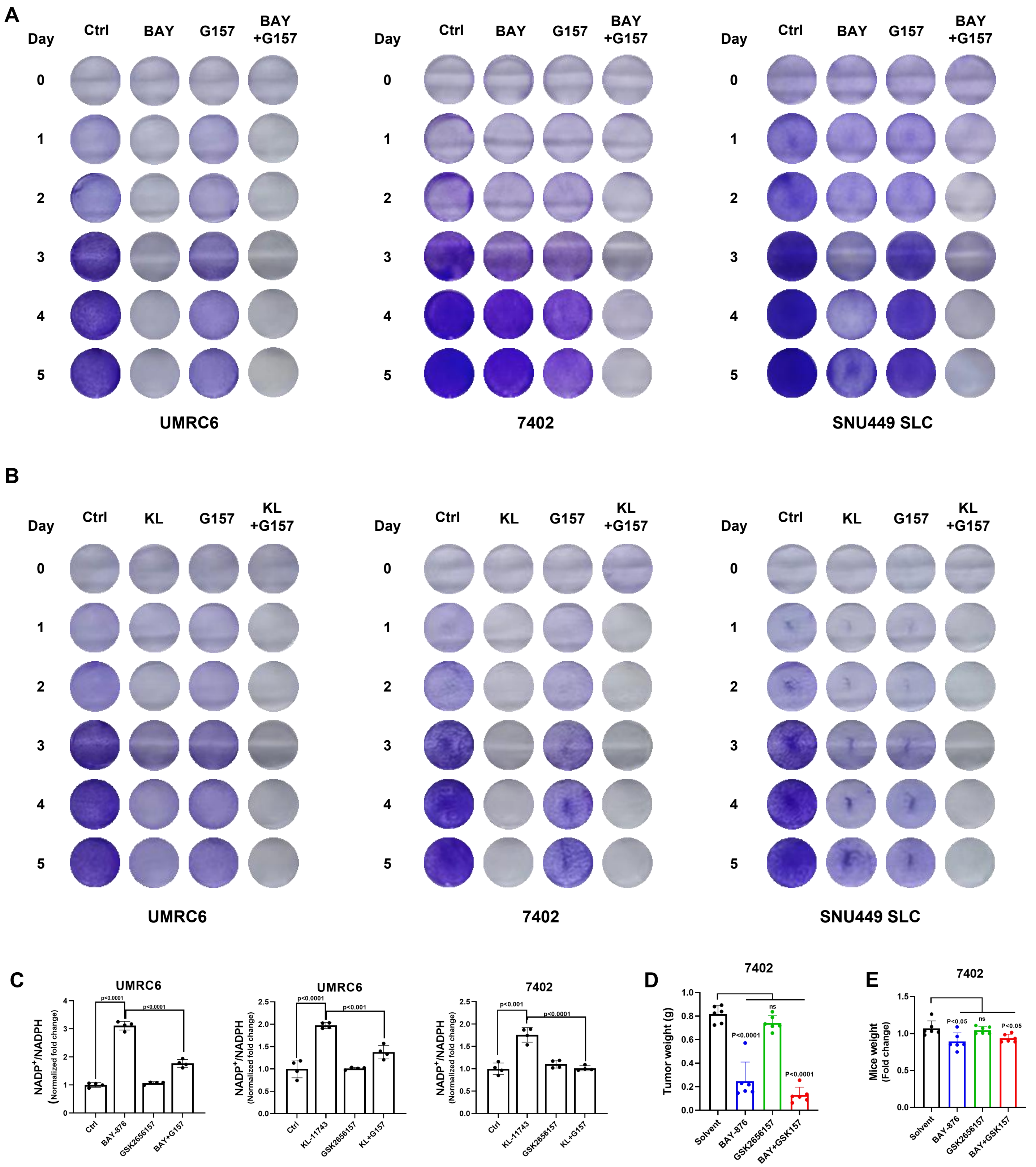
A
Day
Ctrl
BAY
G157
BAY +G157
0
1
2
3
4
5
UMRC6
7402
SNU449 SLC
B
Day
Ctrl
KL
G157
KL +G157
0
1
2
3
4
5
UMRC6
7402
SNU449 SLC
C
UMRC6
NADP⁺/NADPH (Normalized fold change)
p<0.0001
p<0.0001
Ctrl
BAY-876
GSK2656157
BAY+G157
UMRC6
NADP⁺/NADPH (Normalized fold change)
p<0.0001
p<0.001
Ctrl
KL-11743
GSK2656157
KL+G157
7402
NADP⁺/NADPH (Normalized fold change)
p<0.001
p<0.0001
Ctrl
KL-11743
GSK2656157
KL+G157
D
7402
Tumor weight (g)
P<0.0001
ns
P<0.0001
Solvent
BAY-876
GSK2656157
BAY+GSK157
E
7402
Mice weight (Fold change)
P<0.05
ns
P<0.05
Solvent
BAY-876
GSK2656157
BAY+GSK157

Fig S6. Inhibition of ER stress collaborates with GLUT inhibitor to promote disulfidptosis and suppress tumor growth

A-B. Colony formation assay of UMRC6, 7402 and SNU449-SLC cells treated with or without BAY-876 (**A**) or KL11743 (**B**) and G157 for indicated treatment duration. **C.** $NADP^+$/NADPH ratio of UMRC6 and 7402 cells treated with or without BAY-876 (5 μM), KL-11743 (10 μM) and G157 (10 μM) for 4-6 h. **D-E**. Tumor weight (**D**) and fold change of mice weight (**E**) of 7402 xenograft experiment after treatment.

Fig.S7

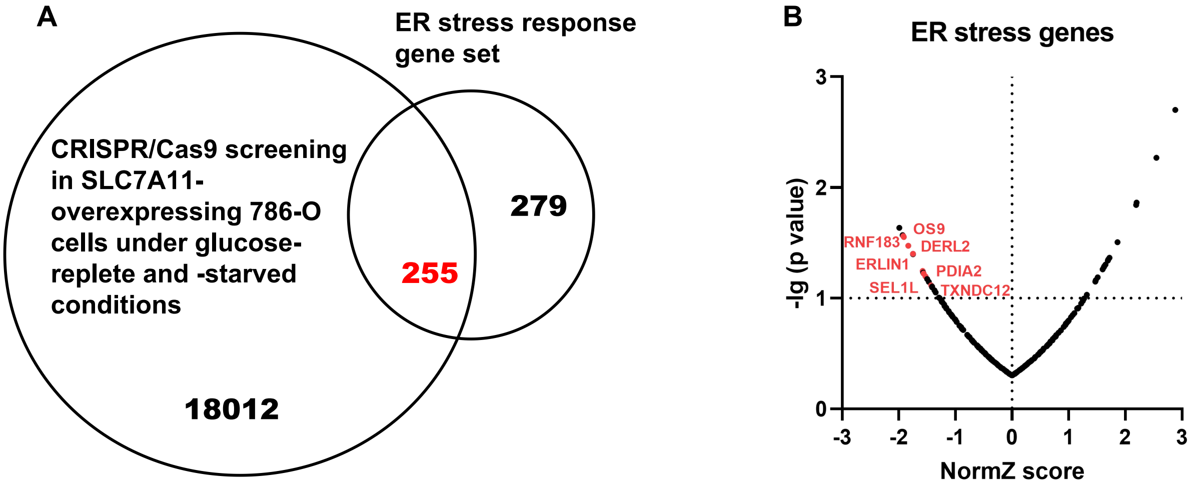

A
ER stress response gene set
CRISPR/Cas9 screening in SLC7A11-overexpressing 786-O cells under glucose-replete and -starved conditions
279
255
18012
B
ER stress genes
-lg (p value)
NormZ score
OS9
RNF183
DERL2
ERLIN1
PDIA2
SEL1L
TXNDC12

Fig S7. Analysis of overlapping genes between disulfidptosis and ER stress response

A. Venn plot of the overlapping genes presented in both the CRISPR/Cas9 screening results and the ER stress response gene list. **B.** Volcano plot of the overlapping genes.

Table S1. LentiCRISPR v2_Forward oligos

sgRNA	Sequence
SLC7A11-sg1	ATGAGCTTGATCGCAAGTTC
SLC7A11-sg2	AAGTATTACGCGGTTGCCAC
MAPK11-sgA	GCGGCTGCTCCGGACATGTC
MAPK11-sgB	CCCTGATGGGCGCCGACCTG
MAPK11-sgC	GGAGCTGAACAAGACCGTGT
MAPK12-sgA	GGGCTCGGGCGCCTACGGCG
MAPK12-sgB	CACCGGCGCTAAGGTGGCCA
MAPK12-sgC	CAGTGGCTTTTACCGCCAGG
MAPK13-sgA	CCCGACGCACGTCGGCAGCG
MAPK13-sgB	GGCCATCGACAAGCGGTCAG
MAPK13-sgC	CCCTGCGCAACTTCTATGAC
MAPK13-sgD	ACCCTTTCAGTCCGAGATCT
MAPK14-sgA	CACAAAAACGGGGTTACGTG
MAPK14-sgB	CTTATCTACCAAATTCTCCG
MAPK14-sgC	TGGACGTTTTTACACCTGCA

Table S2. RT-PCR Primers

Primer	Sequence (5'→3')
hRT-MAPK11-sg1-F	CTTCTGGACGTCTTCACGCC
hRT-MAPK11-sg1-R	GACGATGTTGTTCAGGTCGG
hRT-MAPK11-sg2-F	CGAGGACTTCAGCGAAGTGTA
hRT-MAPK11-sg2-R	GTGGATGTACTTCAGCCCGC
hRT-MAPK13-sg1-F	GGGAGAAGGTGGCCATCAAG
hRT-MAPK13-sg1-R	GCCCAATGACGTTCTCATGC
hRT-MAPK13-sg2-F	AGAACGTCATTGGGCTCCTG
hRT-MAPK13-sg2-R	TCTGCATGAAGGGCATCACC
hGCLC-F	CACCATGGGGCTGCTGTCCCAGGGCTCGCCGCT
hGCLC-R	GTTGGATGAGTCAGTTTTACTTCCAC
